# New *in vivo* avatars of diffuse intrinsic pontine gliomas (DIPG) from stereotactic biopsies performed at diagnosis

**DOI:** 10.18632/oncotarget.15002

**Published:** 2017-02-02

**Authors:** Alexandre Plessier, Ludivine Le Dret, Pascale Varlet, Kévin Beccaria, Joëlle Lacombe, Sébastien Mériaux, Françoise Geffroy, Laurence Fiette, Patricia Flamant, Fabrice Chrétien, Thomas Blauwblomme, Stéphanie Puget, Jacques Grill, Marie-Anne Debily, David Castel

**Affiliations:** ^1^ UMR8203 “Vectorologie & Thérapeutiques Anticancéreuses”, CNRS, Gustave Roussy, Université Paris-Sud, Université Paris-Saclay, Villejuif, France; ^2^ Department of Neuropathology, Hôpital Sainte-Anne, Université Paris V Descartes, Sorbonne Paris Cité, Paris, France; ^3^ Department of Pediatric Neurosurgery, Hôpital Necker-Enfants Malades, Université Paris V Descartes, Sorbonne Paris Cité, Paris, France; ^4^ UNIRS, Neurospin, I2BM, Direction de la Recherche Fondamentale, Commissariat à l’Energie Atomique et aux Energies Alternatives, Gif-sur-Yvette, France; ^5^ Institut Pasteur, Histopathology and Animal Models, Université Paris V Descartes, Sorbonne Paris Cité, Paris, France; ^6^ Département de Cancérologie de l’Enfant et de l’Adolescent, Gustave Roussy, Université Paris-Sud, Université Paris-Saclay, Villejuif, France; ^7^ Université d’Evry-Val d’Essonne, Evry, France

**Keywords:** brain tumor model, child, tumor-initiating cell, infiltrative midline glioma with histone H3-K27M mutation, bioluminescence

## Abstract

Diffuse Instrinsic Pontine Glioma is the most aggressive form of High Grade Gliomas in children. The lack of biological material and the absence of relevant models have hampered the development of new therapeutics. Their extensive infiltration of the brainstem renders any surgical resection impossible and until recently biopsies were considered not informative enough and therefore not recommended. Thus, most models were derived from autopsy material. We aimed to develop relevant *in vivo* DIPG models that mimic this specific disease and its molecular diversity from tumor material obtained at diagnosis. Eight patient-derived orthotopic xenograft models were obtained after direct stereotactic injection of a mixed cell suspension containing tumor cells and stromal cells in the brainstem or thalamus of nude mice and serially passaged thereafter. In parallel, we developed 6 cell-derived xenograft models after orthotopic injection of tumor-initiating cells cultured from stereotactic biopsies. Cells were modified to express luciferase to enable longitudinal tumor growth monitoring, and fluorescent reporter proteins to trace the tumor cells in the brain.

These models do not form a tumor mass, they are invasive, show the H3K27 trimethylation loss *in vivo* and the tumor type diversity observed in patients in terms of histone H3 mutations and lineage markers. Histological and MRI features at 11.7 Tesla show similarities with treatment naïve human DIPG, and in this respect, both direct and indirect orthotopic xenograft looked alike. These DIPG models will therefore constitute valuable tools for evaluating new therapeutic approaches in this devastating disease.

## INTRODUCTION

Brain tumors are the leading cause of cancer-related mortality and morbidity in children, adolescents and young adults. With the refinement of the diagnostic criteria, gliomas now represent the most frequent type of malignant brain tumors [1]. Diffuse intrinsic pontine glioma (DIPG) is one of the most frequent pediatric high-grade glioma. The peak of onset is between 6 and 9 years of age although adolescents and young adults may be affected [2], and the median overall survival of DIPG patients is below 1 year after diagnosis [3]. Because of its infiltrative phenotype and location in an eloquent area of the brainstem, the tumor is unresectable. Diagnosis is usually made on a typical magnetic resonance imaging (MRI) appearance associated with a short clinical history (<6 months). Radiation therapy is only transiently efficient and classical chemotherapy is generally not effective [4, 5].

The recent identification in midline pediatric glioma and in particular in DIPG, of a specific point mutation in *H3F3A, HIST1H3B/C, HIST2H3A/C* histone genes –leading to the replacement of a lysine by a methionine at position 27, has set the basis for a molecular definition of these gliomas [6–8]. This H3-K27M mutation impairs the function of polycomb repressive complex 2 and leads to a global decrease of H3K27 trimethylation in 95% of DIPG samples, reflecting a profound change in the epigenetic landscape of the tumor cells [9–11]. This alteration was chosen to define a new tumor entity in the WHO 2016 classification of brain tumor corresponding to *Midline infiltrative tumors with H3-K27M mutation*, and the associated loss of H3K27 trimethylation could be considered as a driving oncogenic event [12].

If autopsies and stereotactic biopsies of DIPG have recently shed some light on the possible driver mutations, the lack of biological material from DIPG also hampered the development of relevant models mimicking the disease that are crucial to better understand the underlying biology and to evaluate potential new therapeutics [6, 7, 9]. This led some research groups to study the *in vivo* oncogenesis of the tumor through different genetically-engineered mouse models (GEMM) [13, 14]. However, these models are based on the introduction of a limited number of alterations, which are not always found in primary DIPG tumor as for example *PDGFB* overexpression to mimic *PDGFRA* amplification that is especially observed at autopsy. Consequently, it can be questioned whether these models would best recapitulate the disease or if alternative models have to be generated to complete the set of tools to explore DIPG biology.

An alternative to the aforementioned mouse models is the development of cell-derived orthotopic xenograft (CDOX) and patient-derived orthotopic xenograft (PDOX) from patient material. CDOX models were generated by stereotactic injections of cultured cells carrying the histone H3-K27M driver mutation established from DIPG autopsies [15]. More recently, a group successfully developed one PDOX model after direct injection of autopsy DIPG tumor cells [16]. Both types of models showed an infiltrative phenotype, an important characteristic of these neoplasms. However, the biology of the tumor at autopsy may have been substantially altered by the treatment, especially radiotherapy, as shown by the accumulation of new mutations in comparison with biopsy samples, precluding its relevance compared to the tumor at diagnosis. Indeed, genomic alterations in DIPG are more abundant and qualitatively different at diagnosis and at autopsy. The average mutation rate detected at the time of the autopsy is 3.12 somatic mutations per megabase [17] while it is 0.76 at diagnosis [18]. With respect to the type of genomic aberrations, *PDGFRA* gain/amplification are twice more frequent in autopsy samples [17, 19, 20] than they are at diagnosis [18, 21]. Similarly, *ATRX* mutations are rare at diagnosis (absent from the sequencing data of Taylor *et al*. [18]) while they are more common at the time of the autopsy [17]. *ACVR1* mutations were described in all autopsy H3.1-K27M tumors [7] while they were not present in every H3.1-K27M tumors at diagnosis [8]. Such pre-clinical models might thus have a distinct resistance to drugs compared to a treatment-naïve tissue, as well as a potentially altered invasive and migratory phenotypes.

Systematic stereotactic biopsies were re-introduced as a part of the histopathological and molecular diagnosis of DIPG [3]. We took advantage of this treatment-naïve biological material for pre-clinical model establishment representing the disease at the earliest stages possible. The aim of our study was to establish a variety of DIPG xenografts to cover the heterogeneity of DIPG encountered in patients. This is crucial to be able to understand the behavior of the different subtypes of DIPG and generalize the findings from new therapeutics testing. Two methods, direct or indirect xenografting, were tested. Biopsies were either directly xenografted in the thalamus or pons of nude mice, or in parallel cultured *in vitro*, labeled and subsequently injected in the pons of nude mice. These 2 types of pre-clinical models are valuable tools to assess new approaches in this incurable disease as they reflect the primary biopsy in terms of histologic features and expression of classical histopathology markers as well as they reflect the inter-biological differences between patients.

## RESULTS

### Description and processing of the stereotactic biopsy samples

During the course of the study, eighteen patients diagnosed with DIPG were biopsied by stereotaxy (Table 1). This cohort of DIPG has a balanced sex distribution with 9 males and 9 females, a median age at diagnosis of 6 years, and 14/16 children died within the first 2 years after diagnosis (1 patient still alive and 1 was lost to follow-up). Moreover, in all primary tumors, immunohistochemistry analysis showed the expression of a histone H3-K27M mutated protein and the H3K27 trimethylation loss as previously defined as hallmarks of DIPG [8].

**Table 1:** Summary of the molecular & clinical characteristics of DIPG patients and observed tumorigenicity in the corresponding murine models

Patient ID	Histone H3 mutation status	TP53 mutation status	ACVR1 mutation status	Patient			TIC	PDOX model		CDOX model
				Gender	Age	Survival (days)		Thalamus	Pons	Pons
NEM270	H3F3A K27M	R273C	WT	M	10.2	313	Y	-	-	0/10
NEM271	HIST1H3B K27M	WT	Q207E	F	4.4	499	Y	-	-	0/10
NEM273	HIST1H3B K27M	WT	G328E	M	4.6	279	Y	-	-	3/4
NEM276	H3F3A K27M	*N.D*.	*N.D*.	F	6.2	287	Y	-	-	0/10
NEM284	H3F3A K27M	W91*	WT	F	9.3	105	N	0/2	-	-
NEM285	H3F3A K27M	A159V	WT	M	7.1	241	Y	20/22	3/3	5/5
NEM289	HIST2H3A K27M	W146*	WT	M	4.7	262	Y	27/29	8/8	5/5
NEM290	H3F3A K27M	R175H	WT	F	11.6	81	Y	39/40	10/12	25/26
NEM292	H3F3A K27M	P151T	WT	F	5.1	152	Y	-	-	21/26
NEM323	H3F3A K27M	C96Y	WT	M	5.8	269	Y	0/2	-	-
NEM324	HIST1H3B K27M	*N.D*.	*N.D*.	M	1.4	*Alive*	Y	0/2	0/1	-
NEM325	H3F3A K27M	WT	WT	F	5.5	101	Y	4/7	9/9	0/5
NEM328	HIST1H3B K27M	WT	G328V	F	3.5	289	Y	11/12	9/12	5/5
NEM335	H3F3A K27M	R248Q	WT	M	6.2	575	Y	10/13	3/6	0/5
NEM336	HIST1H3B K27M	WT	G328V	M	4.1	*536*	Y	0/2	0/1	-
NEM341	H3F3A K27M	WT	WT	F	8.3	*L.T.F*.	Y	0/2	0/1	-
NEM347	H3F3A K27M	R273C	WT	M	9.1	294	Y	5/5	4/4	-
NEM353	H3F3A K27M	WT	WT	F	6.5	*385*	Y	2/2	1/1	-

For each DIPG patient, the Histone H3, TP53 and ACVR1 mutation status are displayed, together with the demographic and survival data. In the right part of the table, xenografting data are summarized for both direct (PDOX) and indirect (CDOX) strategies. The tumorigenicity is presented as the number of mice presenting tumors over the total number of injected mice. Tumor initiating cells (TICs) that could be successfully cultured from patient biopsies are indicated.

N.D: not determined; L.T.F: lost to follow-up; WT: wild-type; Y/N: yes/no.

Sanger sequencing or Whole Exome Sequencing identified a histone H3 gene mutated in the codon of Lysine 27 in all the tumor samples, resulting in a Lysine to Methionine substitution in either H3F3A for 13/18 of cases (72%), HIST1H3B for 4/18 cases (22%), or HIST2H3A/C for the remaining case (6%) (Table 1 and Supplementary Figure 1).

### Establishment of cell-derived orthotopic xenograft models in the brain of Swiss/nude mice

**Figure 1:**
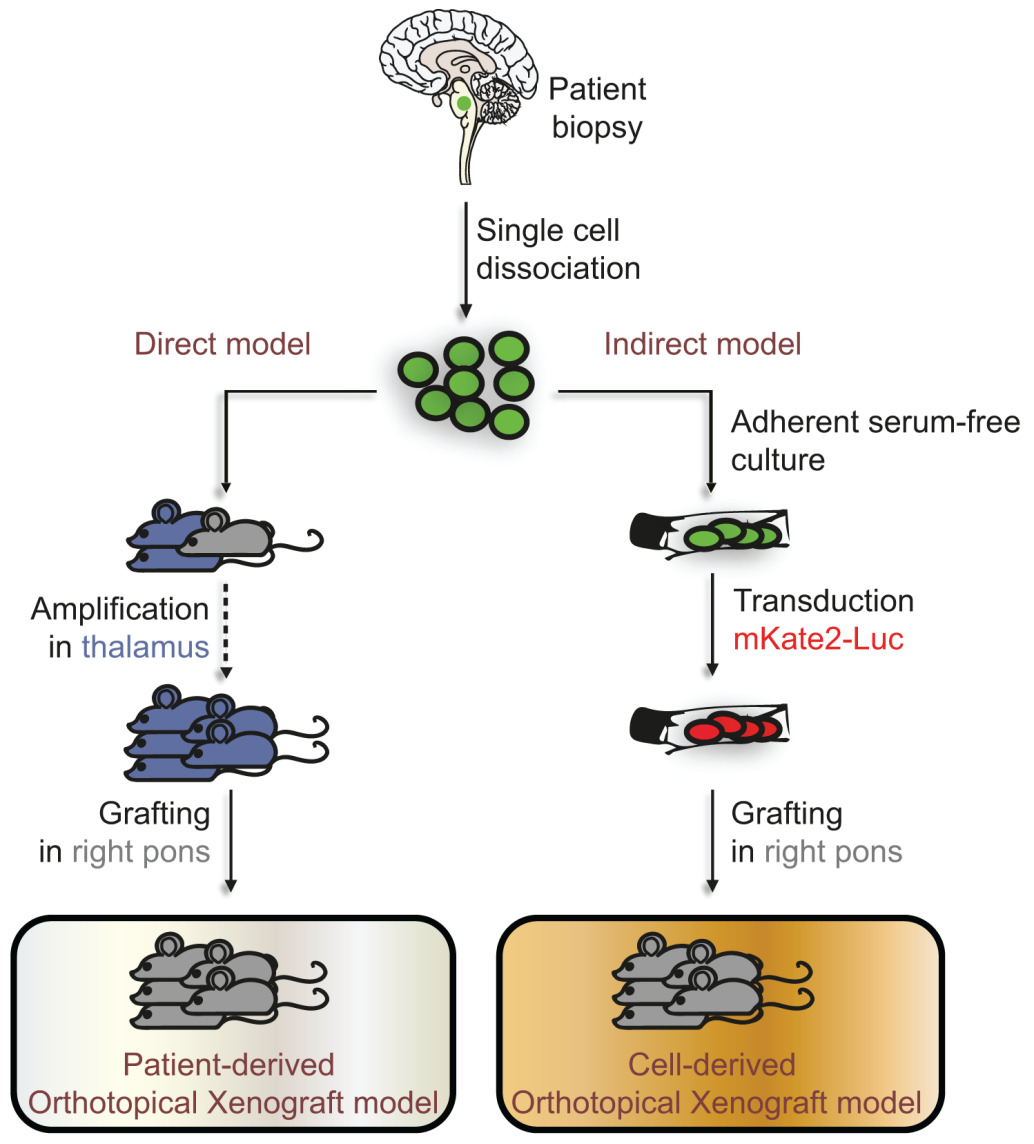
Direct and indirect models developed to study DIPG *in vivo* Tumors originating from the pons of DIPG patients were biopsied and dissociated into a single-cell suspension. Whenever possible, half of the cells were used in the direct model for xenografting into the thalamus of 2 nude mice (in blue), and for latest sample also in the pons of one nude mouse (in grey), constituting the patient-derived orthotopical xenograft models (PDOX). Once a mouse developed neurological symptoms or lost weight, it was sacrificed and the dissociated tumor re-implanted into the thalamus of several other mice to allow the amplification of the tumor cell pool. This method allows the *in vivo* expansion of cancer cells as well as their banking. After 3 successive passages, mice are grafted in the pons. In parallel to the direct injection into the brain, the remaining half of the cell suspension obtained from biopsies was cultured in adherent serum-free conditions. After several passages required for banking, the resulting tumor-initiating cells (TICs) were transduced to express the mKate2 and Firefly Luciferase proteins, and then grafted into the pons of nude mice. These indirect models constitute the cell-derived orthotopical xenografts (CDOX). Green-colored and red-colored cells on the scheme represent the mutated cells found in the biopsy and the mutated cells after transduction of the mKate2-Luc construct, respectively.

Half of the dissociated primary tumor was used to develop *in vitro* cellular models (Figure 1). All eighteen biopsies but one led to primary cell proliferation under these conditions (Table 1). The resulting tumor-initiating cells (TICs) were transduced and xenografted around passage 10 into the pons of 5 nude mice, to establish CDOX. Transduction did not alter the proliferation rate and morphology of TICs (data not shown). For the 4 initial TICs grafting experiments, we injected tumor cells in parallel in five distinct NOD scid gamma (NSG) and nude mice. The take rates were similar in both mice strains with only 1/4 TICs that was tumorigenic.

In total, 11 distinct TICs were xenografted, leading to tumor development in 6 cases and no tumor growth in 5 cases. Consequently, the success rate of CDOX model development can be currently evaluated around 54.5%. Among the six CDOX models that gave rise to tumor, the overall take rate was 90% (n=64/71), taking into account all the injected mice at all passages.

### Successful establishment and long-term maintenance by *in vivo* serial transplantation of patient-derived orthotopic xenograft models in the brain of Swiss/Nude mice

In parallel to the indirect model, as of NEM284, the second half of the single-cell suspension obtained from the patient biospy was directly grafted into the thalamus of several nude mice to allow the expansion of the tumor *in vivo* (Figure 1). For the 8 most recent samples, the tumor cell suspension was also injected in the pons. Once a tumor developed, as suspected when mice became symptomatic, brains were dissected and divided in two following the sagittal plane. Tumor transplantation and banking were performed using the injected side containing most of tumor cells. Histological plus immunohistochemical analyses were performed on the contralateral side to confirm the presence of tumor cells on the same brain. These serial transplantations were repeated until passage 3 in order to significantly amplify *in situ* the initial pool of cancer cells and allow for sample banking. For subsequent passages, cells were xenografted into the right part of the pons of nude mice (Supplementary Table 1).

In total, 8 out of 13 (61.5%) DIPG biopsies were successfully xenografted. In these cases, all replicate mice showed a tumor at first passage. Efficient transplantation from mouse to mouse was achieved, and the rate of tumorigenicity for subsequent passages remained high (91% and 84% for thalamic and pontine injection, respectively). Also, transplanting frozen cells did not significantly decrease the take rate after injection in both locations (85,7% with n=21 and 100% with n=7 for thalamic and pontine xenograft, respectively). However, the survival time was increased in 3 out 5 cases (*P*<0.01, T-test; Supplementary Table 1).

### Survival time comparison between patients and CDOX / PDOX models

Survival time of mice xenografted in the pons varied greatly between the different models, from 80 ±12 days to 299 ±12 days for PDOX, and from 67 ±6 days to 267 ± 28 days in the case of CDOX (Table 2). However, CDOX were highly reproducible with respect to the time to symptoms outbreak in a given model (Table 2). Also, the time to reach end-points tended to be more similar from mouse to mouse for a particular model at a given passage in the case of PDOX models (Supplementary Table 1). Moreover, survival data were overall similar between the subsequent passages of PDOX, with the exception of the first grafting (P0) in several cases.

**Table 2:** Biological properties and histologic features of primary tumor and corresponding *in vitro* and *in vivo* models

Patient ID	Sample Type	Histone H3 mutation status	Mitotic index	Extracellular edema		Doubling time (days)	Max. passage reached	Survival time (days)	Comparison of CDOX and PDOX survival
				Location	Grading				
**NEM273**	Patient	H3.1-K27M	43.7 ±8.3	Pericellular & along axons	2	-	-	279	-
	TICs	H3.1-K27M	N.D.	-	-	2.79	10	-	
	CDOX	H3.1-K27M	25.9 ±4.2	Along axons	3	11.7 ±1	3	239 ±19	
**NEM285**	Patient	H3.3-K27M	24.5 ±0.10	Along axons	1	-	-	241	-
	TICs	H3.3-K27M	N.D.	-	-	2.39 ±0.2	10	-	
	CDOX	H3.3-K27M	N.D.	N.D.	N.D.	15.2 ±1.1	N.D.	199 ±25	N.S (*P=0.25*)
	PDOX	H3.3-K27M	N.D.	N.D.	N.D.	N.D.	4	123 ±6	
**NEM289**	Patient	H3.2-K27M	16.8 ±1.9	Pericellular & along axons	2	-	-	262	-
	TICs	H3.2-K27M	N.D.	-	-	2.15 ±0.17	10	-	
	CDOX	H3.2-K27M	19.6 ±4.5	Pericellular	1	19.1 ±1.1	N.D.	249 ±21	N.S (*P=0.0625*)
	PDOX	H3.2-K27M	N.D.	Pericellular	2	N.D.	4	102 ±9	
**NEM290**	Patient	H3.3-K27M	18.7 ±5.9	Pericellular	2	-	-	81	-
	TICs	H3.3-K27M	N.D.	-	-	2.06 ±0.36	15	-	
	CDOX	H3.3-K27M	20.2 ±4.4	Pericellular	1	10.9 ±1.4	1	135 ±4	** (*P=0.0058*)
	PDOX	H3.3-K27M	20.6 ±9.4	Along axons	1	N.D.	5	80 ±12	
**NEM292**	Patient	H3.3-K27M	24.7 ±17.9	Pericellular	1	-	-	152	-
	TICs	H3.3-K27M	N.D.	-	-	1.92 ±0.06	15	-	
	CDOX	H3.3-K27M	27.4 ±4.8	Pericellular & along axons	3	7.4 ±0.4	N.D.	67 ±6	
**NEM325**	Patient	H3.3-K27M	25.0^#^	Pericellular & along axons	3	-	-	102	-
	TICs	H3.3-K27M	N.D.	-	-	3.57 ±0.08	4	-	
	PDOX	H3.3-K27M	24.4 ±4.7	Pericellular & along axons	2	N.D.	1	299 ±12	
**NEM328**	Patient	H3.1-K27M	50.0^#^	Pericellular	3	-	-	289	-
	TICs	H3.1-K27M	N.D.	-	-	2.66 ±0.33	10	-	
	CDOX	H3.1-K27M	N.D.	Pericellular & along axons	3	19.4 ±4.4	N.D.	267 ±28	N.S (*P=0.125*)
	PDOX	H3.1-K27M	17.1 ±5	Pericellular	2	N.D.	3	179 ±32	
**NEM335**	Patient	H3.3-K27M	30.0^#^	N.D.	N.D.	-	-	575	-
	TICs	H3.3-K27M	N.D.	-	-	3.6	4	-	
	PDOX	H3.3-K27M	28.3 ±5.1	Pericellular	2	N.D.	3	130 ±4	
**NEM347**	Patient	H3.3-K27M	40.0^#^	Along axons	2	-	-	294	-
	TICs	H3.3-K27M	N.D.	-	-	3.42	2	-	
	PDOX	H3.3-K27M	N.D.	Pericellular & along axons	2	N.D.	2	121 ±4	
**NEM353**	Patient	H3.3-K27M	14.7 ±3.5	Pericellular & along axons	3	-	-	385	-
	TICs	H3.3-K27M	N.D.	-	-	1.97 ±0.48	4	-	
	PDOX	H3.3-K27M	N.D.	Pericellular	2	N.D.	1	81 (P0)	

Genotypes of H3 genes resulting from either Sanger or WES sequencing was summarized for all samples. Mitotic indexes were computed using the percentage of MIB-1 positive cells over total nuclei. When scans were not available for primary tumors, data were extracted from the pathologist reports. The extracellular localization of edema was indicated and its magnitude was graded by the pathologist using a scale ranging from 0 to 3. The maximum passage reached with TICs in culture was indicated, as well as the maximum serial transplant performed in mice. Survival time of xenografted mice was estimated only after pontine injection. Differences in survival time between CDOX & PDOX for each model and tumor location were statistically tested (**P<0.01, T-test).

N.D: not determined; N.S: not significant.

We then thought to compare the survival time in mouse models and corresponding patients. Interestingly, we observed a statistically significant correlation between patients and the six CDOX presenting tumors (r=0.839; *P=*0.037; Pearson correlation test). In contrast, neither a correlation nor a trend was found for PDOX models (r=-0.24, *P*=0.61). Lastly, comparing the two kinds of models on the four available pairs, we noticed that the survival time was always shorter for PDOX than for CDOX models from one particular patient. This however appeared statistically significant only for NEM290 (Table 2).

### PDOX and CDOX models display hallmarks of primary human DIPG

For all type of xenografts that showed tumor development, mice presented brutal weight loss (>20% in few days) and/or neurological symptoms onset such as limb weaknesses, impaired motility or tilted head.

First, we assessed the maintenance of histone H3-K27M mutation –present in the human primary tumor, in cultured TICs before transplantation and in murine tumors by Sanger sequencing (Supplementary Figure 1). Single Tandem Repeats (STR) analyses performed in parallel on patient blood samples and on the corresponding mouse tumors confirmed the unique genetic identity of all xenografts (Supplementary Table 2) and the human origin of the tumors that developed in mice.

**Figure 2:**
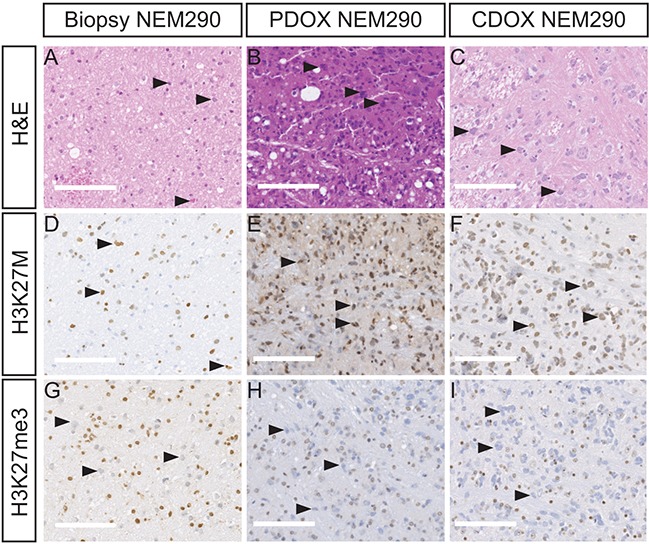
PDOX and CDOX NEM290 models display DIPG hallmarks Representative results of Hematoxylin & Eosin colorations **A-C.** and IHC staining for H3-K27M **D-F.** or H3K27 trimethylation mark **G-I.** in the tumor biopsy NEM290 and the corresponding PDOX and CDOX. In all three cases, H&E and IHC allowed the detection of abnormally-shaped nuclei, tumor cells positive for the histone mutation K27M as well as cells losing the trimethylation marks at position 27 of histone H3, thus showing the conservation of the DIPG hallmarks in the PDOX and CDOX mouse models. Arrowheads in the different panels highlight example of characteristic tumor cells. (Scale bars: 125 μm).

**Figure 3:**
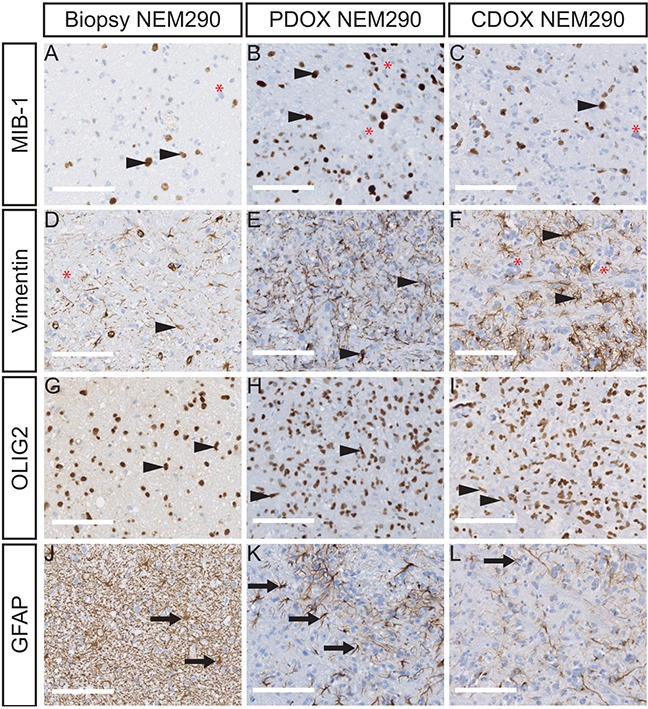
PDOX and CDOX NEM290 models harbor consistent histopathological features of the DIPG tumor of origin Representative microphotograph of NEM290 tumor biopsy and sagittal sections of PDOX & CDOX corresponding models labelled for MIB-1 **A-C.**, Vimentin **D-F.**, OLIG2 **G-I.** and GFAP **J-L.** The staining for MIB-1 and Vimentin show both positive and negative tumor cells in all samples, reflecting the phenotypic tumor heterogeneity. OLIG2 positivity is pervasive in both normal and tumor cell compartments. High GFAP levels were mainly detected in reactive astrocytes in all samples, but a stronger staining on basal human stromal cells was observed in the biopsy sample. Arrowheads indicate positive tumor cells. Red asterisks indicate negative tumor cells. Arrows indicate normal parenchymal cells. (Scale bars: 125 μm).

Additionally, the H&E staining allowed the detection of tumor cells with dense and irregular nuclei in the brainstem both of the patient and mice samples (Figure 2A-2C and Supplementary Figure 2). Moreover, the detection of the K27M-mutated histone H3 by IHC showed a strong positivity throughout the pons in the 12 xenograft models we could test (Figure 2D-2F, Supplementary Figures 3A-3B and 4), as well as the associated H3K27 trimethylation loss (Figure 2G-2I and Supplementary Figure 3C-3D). The use of a human-specific MIB-1 antibody confirmed the presence of proliferating cells of human origin in all tested xenograft samples (10/14 models) in the same area as K27M mutated cells. We observed a mixture of tumor positive-cells and normal negative-cells in mice brains (Figure 3A-3C and Supplementary Figure 5). Of note, the mitotic indexes appeared mostly similar in the xenograft models as compared to the primary biopsies (Table 2).

More importantly, these staining highlighted the presence of intermingled tumor and normal stromal cells, thus confirming that CDOX and PDOX models recapitulate the main histological hallmark of DIPG with an extensive infiltration of the brain parenchyma by tumor cells without formation of a tumor mass and without neovascularization. Likewise, mouse xenografts displayed moderate to high extracellular edema, as observed in the primary tumor, pericellular or along the axonal stream (Table 2 and Supplementary Figure 2).

### The immunophenotype of the human tumor is conserved in mouse xenograft models

The additional labeling of proteins currently used for DIPG diagnostic purposes were performed in parallel on primary tumors and both NEM290 and NEM292 xenograft models. We first stained the intermediate filament Vimentin, a known marker of neural stem cells and early glial progenitors, also detected in gliomas and reactive astrocytes, but barely expressed in the normal brain [22] (Figure 3D-3F). We observed in the models an expression of Vimentin in tumor cells recapitulating the primary tumor and confirming the glial phenotype of the cancer cells. Strikingly, not all tumor cells expressed the marker both in the primary tumor and the models, pointing to a phenotypical intra-tumor heterogeneity. The oligodendrocyte precursor marker OLIG2, which is common to all DIPG, was robustly detected in most if not all tumor cells in primary human and mouse xenograft DIPG samples (Figure 3G-3I). Lastly, the staining of the astrocytic intermediate filament GFAP antibody labeled all reactive and stromal astrocytes in the biopsy. Because of the higher antibody affinity for the human protein, it only detected cells highly expressing GFAP, *i.e*. mouse reactive astrocytes, in the infiltrated parenchyma (Figure 3J-3L). Outside of these areas, the staining was mostly negative (except in the subpial space; data not shown). These results indicate that we could recapitulate the reactive stroma in the xenograft model as observed in primary human tumors. Similar results were obtained in the case of NEM292 (Supplementary Figure 3). Overall, all these staining confirmed the infiltrative nature of an oligo-astrocytic neoplasm of human origin without tumor mass formation, that showed identical features of differentiation, biomarker heterogeneity and reactive astrogliosis found in the primary DIPG. Also, all staining showed identical results for both direct (PDOX) and indirect (CDOX) NEM290 xenografting approaches.

### Infiltration of the brain parenchyma and dissemination of DIPG tumor cells

**Figure 4:**
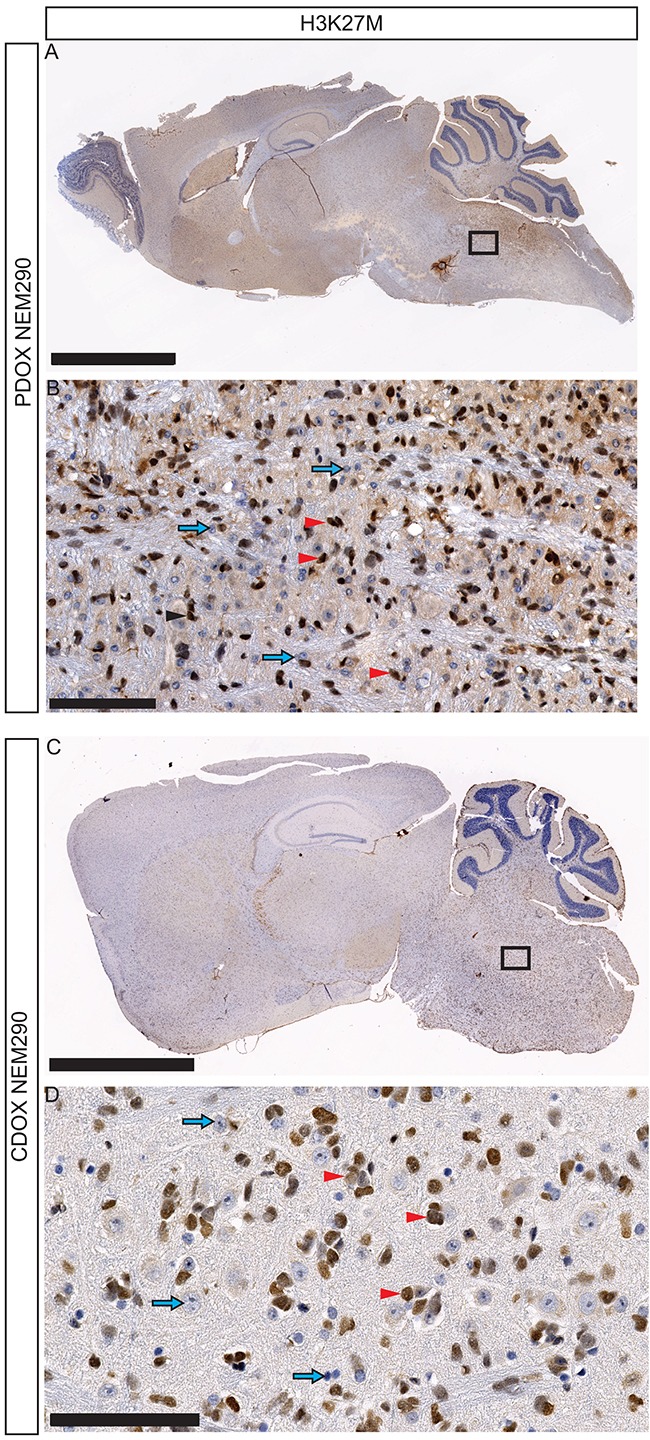
Invasion and dissemination of tumor cells within PDOX and CDOX models of NEM290 **A.** Representative low magnification microphotograph of a sagittal section of PDOX NEM290 stained for H3-K27M mutation (brown nuclei) and **B.** 30X close-up from the square region. **C.** H3-K27M immunostaining of a sagittal section of CDOX NEM290 and **D.** corresponding 30X magnification. An invasion around the injection point in the pons, toward the cerebellum and the rostral axis is observed in both cases, as well as subpial and subventricular dissemination. (Scale bars: **A** and **C**, 2.5 mm; **B** and **D**, 100 μm). Red arrowheads indicate positive tumor cells; blue arrows indicate negative non-tumor cells.

IHC labeling of H3-K27M protein on sagittal sections allowed us to investigate the location of tumor cells in the whole brain. Indeed, H3-K27M positive-cells were detected in the entire pons – beyond the injection site, mixed with unlabeled normal cells in both PDOX and CDOX models (Figure 2D-2F and Figure 4). There was an expansion of the cancer cells without any tumor mass formation thus highlighting a pairing of the infiltration process with the tumor growth.

The full sagittal sections showed that H3-K27M positive-cells were not restricted to the pons but infiltrated massively toward the rostral axis of the brain, the cerebellum, the subpial space, the cortex and the thalamus (Figure 4, Supplementary Figures 6-9). We observed to a lesser extent infiltration in the spinal cord (data not shown). Interestingly, in approximately 30% of injected mice, tumor cells also migrated in the olfactory bulbs through a ventral path along the meninges in lateral hypothalamic and olfactory stream areas (Supplementary Figures 6 and 7).

### Bioluminescence, fluorescence of CDOX models

**Figure 5:**
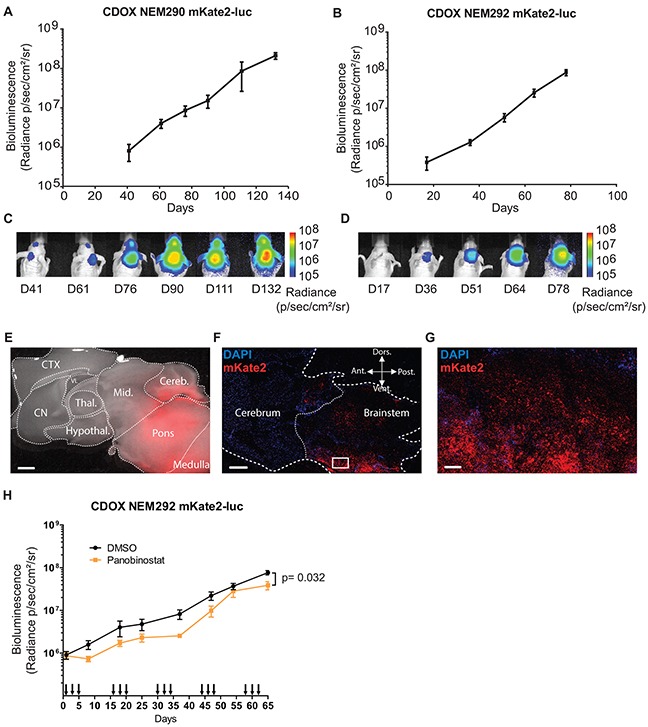
*In vivo* tumor growth can be monitored by bioluminescence and fluorescence **A-B.** Longitudinal follow-up of tumor growth by quantification of the luciferase activity in the pontine region of CDOX NEM290 mKate2-luc and CDOX NEM292 mKate2-luc, respectively. Both models show a robust exponential growth of BLI. Results are represented as mean ±SEM (n=3 and n=5 replicate experiments). **C-D.** Corresponding representative images of bioluminescence in the aforementioned CDOX models. **E.** Overlay of bright-field and mKate2 fluorescence (red) microphotographs of a brain sagittal section of CDOX NEM292 at 5X magnification. Note the extensive infiltration and invasion towards the cerebellum and midbrain. (Scale bar: 1 mm). **F.** Confocal micrograph of a sagittal section of CDOX NEM292 with Hoechst (blue) and mKate2 (red) fluorescence at 10X magnification. (Scale bar: 1mm). **G.** 20X magnification of the square region highlighted in F, showing the infiltration of the parenchyma by tumor cells. (Scale bar: 100 μm). **H.** Preclinical evaluation of the activity of the panobinostat on DIPG tumor growth by longitudinal quantification of the luciferase activity in the pontine region of CDOX NEM292 mKate2-luc after panobinostat (n=7 mice) or DMSO (control vehicle, n=9 mice) injections. Panobinostat was administered I.P., three times a week at 10 mg/kg, every other week. Injections are indicated by arrows on the x-axis. Results are represented as mean ±SEM. *P < 0.05; Statistical permutation test (see methods). CTX: cortex; CN: cerebral nuclei; Thal.: thalamus; Hypothal.: hypothalamus; Mid.: midbrain; Cereb.: cerebellum; VL: lateral ventricle; Ant.: anterior; Vent.: ventral; Dors.: dorsal; Post.: posterior.

A major advantage of CDOX models over PDOX is the possibility to longitudinally monitor disease progression by bioluminescence and fluorescence imaging. We consequently assessed the *in vivo* tumor growth kinetics by recording the bioluminescence (BLI) signal every 15-25 days. The models tested showed an increase of the signal with a doubling time around 10.9 ±1.4 days (n= 3 mice) in the case of NEM290, and 7.4 ±0.4 days (n= 5 mice) in the case of NEM292 (Figure 5A-5D and Table 2). The bioluminescence curves for the 4 others CDOX models are presented in Supplementary Figure 10.

The proliferation rate *in vivo* was always lower compared to *in vitro* TICs, which doubled approximately every 2 or 3 days (Table 2). For CDOX NEM290 and NEM292, the models with the most important number of biological replicates, we observed the appearance of clinical symptoms, and thus the terminal stage of the disease, at the same BLI level (around 10^8^ p/s/cm^2^/sr) in several distinct experiments. Besides, the additional CDOX models showed mostly equivalent BLI at end-stage (10^8^-10^9^ p/s/cm^2^/sr; Supplementary Figure 10).

Moreover, red fluorescence of cells in CDOX models allowed for the detection of the tumor location after brain excision by using macroscopic imaging of the whole brain, highlighting the extensive infiltration of the mouse brain (Figure 5E; Supplementary Figure 11). The tracking of tumor invasion at single cell resolution on slices by confocal microscopy highlighted the migratory stream of tumor cells in the brain (Figure 5F-5G). Also, dissociated tumor cells could be retrieved from the animal samples by FACS for subsequent analyses thanks to their fluorescence.

In order to demonstrate the value of these models to conduct preclinical drug evaluation by bioluminescence monitoring of tumor growth, we performed an *in vivo* evaluation of the effect of panobinostat, a drug known to be effective in DIPG [23]. We could confirm a moderate but statistically significant effect of panobinostat on CDOX NEM292 tumor growth (Figure 5H).

### Blood-brain barrier integrity of CDOX models

Several reports have shown that a proportion of DIPG tumors at diagnosis present a relatively intact and functional blood-brain barrier (BBB) as assessed by MRI contrast enhancement [8, 24]. We thus tried to check the status of the BBB in the DIPG xenograft models. First, looking at hematoxylin & eosin staining, we did not observe a microvascular proliferation that would go along with compact tumor tissue or necrotic areas even at late stage of tumor development (Figure 2 and Supplementary Figure 12). Still, we probed the expression of GLUT1, which loss of expression in endothelial cells can accompany BBB disruption in several different pathophysiological conditions [25]. In both CDOX NEM290 and NEM292, we observed a staining of endothelial cells from vessels and capillaries in the brain parenchyma. However, the region of the brain infiltrated with tumor cells (pons) showed much less intense staining (Supplementary Figure 12). Given these discrepancies, we finally checked for a functional disruption of the BBB by assessing Evans blue dye extravasation after intravenous injection in normal and tumor bearing mice [26, 27]. As expected for control mice, the brain remained unstained, as opposed to other peripheral organs (such as kidney; Supplementary Figure 13A-13M). The same experiment conducted on CDOX NEM290 and NEM292 led to identical results, without evidence of blue dye diffusion in the brain and in particular in the infiltrated pons (Supplementary Figure 13).

### Implanted tumors are detectable by ultra-high field MR-imaging

**Figure 6:**
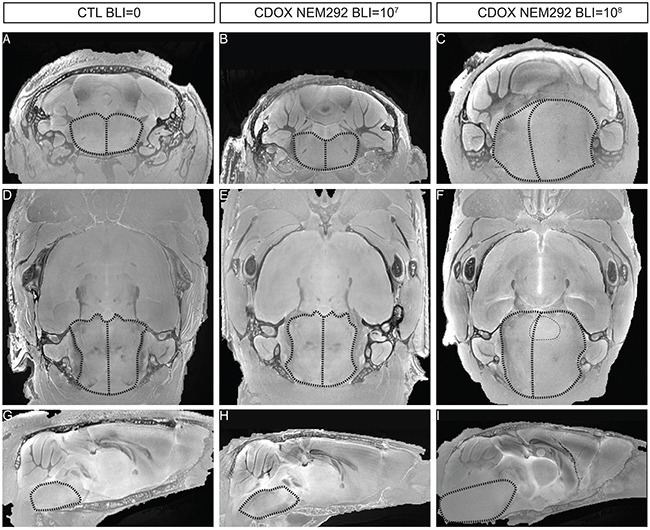
T2-sequence MRI modalities of DIPG are recapitulated in the brainstem of end-stage CDOX models Anisotropic T2-sequence MR-Imaging at 11.2T of a non-grafted control mouse **A, D, and G.**, CDOX NEM292 showing a 10^7^ BLI level **B, E, and H.** and CDOX NEM292 showing a 10^8^ BLI level **C, F, and I.** For visualization, images were focalized on the pons according to the coronal plane **A, B, and C.**, the axial plane **D, E, and F.** and the sagittal plane **G, H, and I.** sections. Anatomical changes with a midline shift and pons hypertrophy were observed for the 10^8^ BLI mouse only. A hyperintensity could also be observed in the pontine region (highlighted with dots in F). Brainstem and midline are depicted by dashed lines.

We finally investigated how MR-imaging modalities of DIPG were recapitulated in CDOX models. Indeed, T2-weighted MRI is one of the only non-invasive methods for DIPG diagnosis, showing an infiltration respecting the pontine fiber tracts and an inflated brainstem in patients. We performed *ex-vivo* T2-RARE (Rapid Acquisition with Relaxation Enhancement) MR acquisition at 11.7 Tesla in 3 different animals: the first showing an intermediate bioluminescence radiance signal at 10^7^ (28 days post-graft), the second in a mouse reaching 10^8^ (77 days post-graft) that we characterized as advanced stage of the disease, and a last non-injected control mouse. Only anatomical changes were observed due to the highly infiltrative nature of the tumor with a shift of the midline to the left in coronal and axial view, much more pronounced in the 10^8^ BLI-positive mouse than in the 10^7^ (Figure 6C, 6F *vs*. 6B, 6E). The inflation of the pons was also more prominent at higher BLI signal on sagittal panels (Figure 6H *vs*. 6I). We also noticed a slight hyperintensity in the right side of the brainstem corresponding to the site of injection on the axial panel (Figure 6F).

## DISCUSSION

We show here that it is possible to derive xenografts from DIPG stereotactic biopsies performed at diagnosis with two distinct methods based either on the direct implantation of tumor cells right after the biopsy or after few passages *in vitro* (PDOX or CDOX, respectively). Tumor behavior in mice recapitulated the natural history of the disease with similar symptoms and a rapid onset after a long silent period [28]. The take rates of the different models using adult nude mice were high –even post-freezing of tumor cells, around 55-60% for both PDOX and CDOX, which is much higher than the take rate of other malignant pediatric tumors in our hands [29, 30]. Our xenografting results using NSG mice are however too limited to firmly conclude that these are not superior to nude mice in terms of take rate –especially for PDOX development. We did not observe differences in tumorigenicity between the direct and indirect xenografting methods (*P*=1; Fisher exact test). Also, xenograft take rate was not linked to the type of histone H3 mutated. We found a significant correlation between patient and CDOX survival, not retrieved in the comparison patient *vs*. PDOX. This discrepancy could originate from distinct number of injected cells between models that could not be controlled before injection in contrast with CDOX models. Besides, we observed that PDOX survival time tend to be shorter than CDOX which could reflect an impact of the stroma and microenvironment injected together with the tumor cells. There are few publications describing xenograft models of DIPG and only limited information is available in the literature on the best way to generate these models [15, 16, 31]. Most research groups inject the tumor cells from autopsies and not from stereotactic biopsies in NSG or nude mouse pups or NOD-scid adult mice, but most take rates and information about serial transplantations are not provided, thus limiting the possibility to compare the different strategies.

Detailed histo-pathological analyses demonstrated that PDOX and CDOX tumors look alike, indicating that *in vitro* selection of tumor-initiating cells (TICs) does not modify the phenotype of the xenografts and that TICs can recapitulate the entire tumor. Remarkably, only tumors of human origin were observed in our PDOX nude models from pre-treatment biospies, contrary to previous published reports using autopsy samples [31].

In depth analysis of the resulting tumors showed that the xenografts retained the phenotype of the patient's tumor in particular the main hallmarks of DIPG, *i.e*. a mostly infiltrative phenotype without any tumor mass, the absence of neovascularization, a functional BBB and the conservation of the H3-K27M mutation identified in patients and the characteristic H3K27 trimethylation loss. In addition, we observed a close conservation in the expression of embryonal and neural differentiation genes (GFAP, OLIG2 or Vimentin), as well as similar proliferation indexes between the models and primary tumors. The phenotypic heterogeneity observed in primary DIPG, more strikingly defined by the presence or absence of Vimentin expression, was recapitulated. Similarly, both cycling and non-cycling tumor cells (*i.e*. MIB-1 negative) were identified in the models as in the patient tumor. Interestingly, the influence of the tumor on brain parenchyma was recapitulated in the mouse as shown by the presence of a reactive astrogliosis and pericellular edema.

A common evolution of the disease is the extensive invasion of DIPG cells in neighboring brain structures, or even in distant location. In late-stage xenograft models, we found such invasion of the pons, cerebellum and thalamus and to a lesser extend the spinal cord. Whole brain observation of our models identified also long distance migration in preferential routes such as subpial and ventricular zones, or metastasis in the olfactory bulbs. However, no particular immuno-phenotypic differences could be observed between invading cells and pontine “resident” tumor cells.

MRI did not allow a proper follow-up of these infiltrative models except at end-stage. In this regards, CDOX models are powerful tools allowing the *in vivo* follow-up by bioluminescence only few weeks after injections. The robustness of BLI increase permits to precisely analyze distinct stage of disease development. Likewise, the additional fluorescent reporter is also particularly convenient to identify the tumor cells and metastases in the fresh tissue, and allows for FACS sorting of the living tumor cells from the animal for further study. Finally, these CDOX models constitute a potent platform, as TICs can be modified using lentiviruses prior to their injection in order to assess the *in vivo* consequences of gene modulation (overexpression or knock-down).

One of the advantage of our models in comparison with the GEMMs is the use of cancer cells presenting from start the genetic consequences of the epigenetic modifications, such as the global trimethylation loss resulting from the H3-K27M alteration that needs a significant number of division before it can be observed, together with the associated mutations landscape [9]. Indeed, it is difficult to recapitulate in GEMMs the temporal succession of genetic alterations occurring during oncogenesis.

Xenografts generated from treatment-naïve diagnostic samples may usefully complement those generated from the autopsies. The former represents the initial stages of the disease, *i.e*. the ideal situation to test new drugs to be incorporated in the initial management of patients. The later represent the end stages of the disease, *i.e*. models that usually display additional alterations resulting from treatments and progression, with potentially more aggressive behavior mirrored by faster growth rates [23]. Autopsy models thus appear more appropriate to test for resistance mechanisms and understand the evolution of the disease by comparison with models derived from biopsies performed at diagnosis. Depending on the biological question, the different types of models now available in the field would interestingly complement each other in order to ease the understanding of the disease and the development of efficient therapeutics.

## MATERIALS AND METHODS

### Tissue collection

All DIPG tumor samples were stereotactically biopsied at diagnosis in Necker-Enfants malades hospital (Paris, France). Informed consent for the translational research program was obtained from the parents or guardian according to the IRB approved protocol (CNIL 1176643). Patient's clinical characteristics are summarized in Table 1. Blood DNA extracted from the blood of patients was banked under the same informed consent.

Immediately in the operating room, one core biopsy sample of the tumor was transferred into DMEM with 100 U/ml penicillin and 100 μg/ml streptomycin (Gibco). Within 24-hours after surgery, biopsies measuring approximately 1 × 4 mm were mechanically dissociated by pipetting into serum-free medium in order to obtain a single-cell suspension. Another sample was directly snap frozen in dry ice and processed later for DNA extraction.

### Tumor-initiating cells (TIC) culture

The dissociated tumor cells suspension was cultured as an adherent monolayer in laminin-coated flask (Sigma) with a medium consisting of NeuroCult NS-A with proliferation supplement (Stemcell technologies), heparin (2 μg/mL, Stemcell technologies), human-basic FGF (20 ng/ml, Peprotech), human-EGF (20 ng/ml, Peprotech), PDGF-AA (10 ng/ml, Peprotech), and PDGF-BB (10 ng/ml, Perprotech). Medium was renewed every 2-3 days and passaging was performed when cells reached a confluence around 70-80%. Doubling time of TICs was evaluated based on proliferation assay recorded by video-microscopy.

### Patient-derived orthotopic xenograft and serial transplant

Single-cell suspension from biopsy was centrifuged and resuspended into 50 μl of serum-free supplemented culture-medium. Female Swiss athymic nude mice were purchased at age 4-6 weeks from the Preclinical Evaluation Platform of Gustave Roussy. Mice were kept under sterile conditions in insulators and received food and water *ad libitum*. For tumor cells implantation, mice were anesthetized by 2.5% isoflurane (1.5 L O_2_/min) and placed into a stereotactic frame. For the first graft, 5 μl of cell suspension was injected into the thalamus of two mice, and for more recent biospies 3 μl of cell suspension was also injected into the pons of a third mouse. After appearance of neurological symptoms or more than 20% body weight loss, mice were sacrificed by CO_2_ inhalation. Brain was removed under sterile conditions and divided in two parts following the sagittal plane. The injected side of thalamus or pons (according to injection coordinates) was isolated and cut to obtain 1mm^3^ pieces using a sterile scalpel, and then enzymatically dissociated using the Tumor Dissociation Kit following the manufacturer instructions (Miletnyi, Germany) and the cell suspension was passed through 100 μm strainers. Thereafter, cells were centrifuged and washed with 1X PBS prior to be resuspended in serum-free supplemented-medium. One fifth of the resulting cell suspension was xenografted into the thalamus or pons of several recipient mice, corresponding to passage 1. Each mouse was injected with the equivalent of 1/20^th^ of the dissociated tumor in 5 μl for thalamic injections and 3 μl for pontine injections. The remaining cells (4/5^th^) were frozen in serum free medium supplemented with 10% DMSO to allow for biobanking. The procedure was repeated until passage 3. At this time, the cells were grafted into the pons of mice in 3 μl of medium. The remaining half of the brain was fixed in Formol/Zinc overnight at room temperature and then processed for IHC.

Stereotactic coordinates used to target the thalamus or the pons were: 3 mm posterior, 2 mm lateral to bregma, 3.5 mm deep, or 1 mm posterior, 1mm lateral to lambda and 5 mm deep, respectively. All *in vivo* experiments were performed under conditions established by the European Community and approved by the CEEA26 Ethic Committee and the French Ministry (approval number: APAFIS#675 and APAFIS#1141).

### Lentiviral transduction

The coding sequences of the fluorescent protein mKate2 [32] and *Photinus pyralis* (Firefly) luciferase were cloned in frame downstream of a synthetic CAG promoter in a pLVX lentiviral vector (Clontech) with a P2A auto-cleavable peptide between them. Lentiviral particles were produced in HEK293T cells using psPax2 and pMD2.g second-generation packaging plasmids (Addgene #12260 and #12259). TICs were transduced around passage 7 for 4 hours with concentrated virus at a multiplicity of infection about 2 and the medium was then renewed.

### Development of cell-derived orthotopic xenograft

TICs within the two passages subsequent to lentiviral transduction were harvested with accutase and resuspended in serum-free supplemented-medium. Three hundred thousand cells were injected into the pons of adult mice in a total volume of 3 μL (*i.e*. 100,000 cells/μl) as described above. Mice were kept under specific pathogen-free conditions in air-filtered cages and received food and water *ad libitum*. Tumor growth was monitored using an IVIS 50 system with charge-coupled device (CCD) camera (PerkinElmer) every 2-3 weeks. Mice were anesthetized as described previously and imaged 10 min after intra-peritoneal injection of luciferin (150 mg/kg body weight, Promega). Radiance signal intensity (normalized photon counts in photons/second/cm^2^/steradian) in the pons region was quantified using Living Image software (Perkin Elmer). Doubling time of the CDOX models was estimated based on bioluminescence exponential curves.

### *In vivo* administration of Panobinotstat

Administration of Panobinostat was performed by intraperitoneal (I.P.) injections of 10 mg/kg at day 1, 3 and 5, every other week, up to 62 days post xenograft. Control mice were injected with identical concentration of vehicle (DMSO). Panobinostat was dissolved as described by Grasso *et al*.,[23] at 70 mg/ml in DMSO, then serially diluted in water to a concentration of 1 mg/ml such that 10 μl per gram of weight (or ∼200μl total) were injected I.P at each administration. Longitudinal tumor growth monitoring was performed by bioluminescence measurement as aforementioned. A statistical permutation test with the ‘compareGrowthCurves’ function of the statmod software package for r (http://bioinf.wehi.edu.au/software/compareCurves) was used to evaluate the significance of differences in the rate of tumor development between the two groups and 10,000 permutations were performed [33, 34]. The initial *P*-values were adjusted for multiple testing correction using Holm's method.

### Immunohistochemical staining

Intracardiac perfusion with 10 mL of 1X PBS was performed in mice to remove blood from the brain and subsequent perfusion of 10 mL Formalin/Zinc (formol 5%; Zinc 3 g/L; sodium chloride 8 g/L; MM-France) was performed for brain fixation with an osmotic pump delivering 7 mL/min. Brains were extracted and fixed for an additional 24 hours in Formalin/Zinc solution at room temperature prior to paraffin-embedding.

Representative formalin-zinc fixed sections were deparaffinized and subjected to a Ventana autostainer (BenchMark XT, Ventana Medical system, Tucson, USA) with routine protocols. Antigen retrieval was performed either with a standard pretreatment protocol including CC1 buffer for MIB-1 (Ki67, 1/100, Dako) and GLUT1 (1/100, ab652 AbCam) or in a semi-automatized system using a microwave antigen retrieval (MicroMED T/T Mega; Hacker Instruments & Industries, Inc., Winnsboro, SC) for 30 minutes at 98°C for OLIG2 (1/500, Sigma), Vimentin (1/750, Dako), GFAP (1/200, Dako), H3K27M (1/1000, Millipore) and H3K27me3 (1/1250, Diagenode). Staining used the RTU Vectastain Universal detection system (Vector laboratories, Burligame, CA, USA). Slides were scanned on a NanoZoomer Digital Slide Scanner (Hamamatsu, Japan). `

Tumor histology was reviewed by a neuro-pathologist (P.V.). Tumors were classified and graded according to the 2007 WHO classification. The quantification of MIB-1 positive cells in PDOX and CDOX models as well as biopsies (when available), was performed on scanned slides. To facilitate the quantification, the whole pons and medulla were sectioned into approximately 20 pictures of 20X magnification. Then, a macro was developed on Fiji (2.0.0) to specifically quantify the number of blue nuclei (negative cells) and the number of brown (positive) nuclei. The ratio of positive cells among the total number of nuclei found was then calculated for each picture and results are given as mean +/− SD. When MIB-1 staining of patient tumors were not available, data were extracted from the pathologist report.

### Genotyping and STR analysis

Tumor DNA was extracted either from TICs, excess of ungrafted cells during PDOX transplant and FFPE tissue sections for CDOX using the QIAamp DNA Mini kit following manufacturer instructions (Qiagen). Histone H3 genes were amplified by PCR as previously described [8]. PCR products were analyzed by direct Sanger sequencing. Short tandem repeats (STR) analyses on patient blood, xenografted tumor or TICs DNA were outsourced to Eurofins Genomics (Germany).

### Fluorescence microscopy

After dissection and fixative perfusion, brains were placed onto a sagittal brain matrix and a 2 mm-slice was cut from the middle line using razor blades. This slice was placed in a 1X PBS solution containing 10 ng/mL of Hoechst for 50 min. Thereafter, the slice was washed in a 1X PBS solution and put either on a dish for macroscopic imaging (Leica MZFLIII) or in a glass-bottom dish for confocal imaging (Leica SP8).

Serial images taken on the fluorescent macroscope were stitched with MosaicJ on Fiji to allow for the reconstruction of the whole brain. Thereafter, red channel corresponding to the mKate2 fluorescence, and bright-light channel were merged with Fiji to allow for the detection of tumor infiltrated structures.

### *Ex vivo* Magnetic Resonance Imaging (MRI) of mouse brains

Mice were anesthetized with sodium pentobarbital (100–150 mg/kg, intraperitoneally), followed by intra-cardiac perfusion with Formol/Zinc solution (see above). After perfusion, each mouse head was harvested while maintaining brain integrity into the skull, and stored in Formol/Zinc solution at 4°C. Before MRI acquisition, the head was washed into PBS for 48 hours to remove the fixation solution, and then placed into a custom-built MRI compatible tube filled with Fluorinert FC-40, a magnetic susceptibility-matching fluid (3M, Saint Paul, USA).

*Ex-vivo* MRI acquisitions were performed on an 11.7T preclinical scanner (Bruker, Ettlingen, Germany) equipped with a cryo-coil dedicated to mouse brain. T_2_-weighted anatomical images were acquired using a 3D TurboRARE (Rapid Acquisition with Refocused Echoes) sequence: TR = 2 000 ms, TE = 6.75 ms, RARE factor = 8, effective TE = 20.25 ms, number of averages = 6, acquisition time = 23 h. The images dimension was set to 328 × 288 × 168 voxels, with a field-of-view of 16.4 × 14.4 × 8.4 mm^3^, which provided a high isotropic spatial resolution of 50 × 50 × 50 μm^3^ for the mouse brain. Such very high isotropic spatial resolution should limit partial volume effects and increase the sensibility to detect morphometric changes. T_2_-weighted anatomical images were reconstructed from raw data using homemade routines written in Matlab (Mathworks, Natick, USA). A specific algorithm was used to correct signal heterogeneities induced by the surface coil [35] and 3D visualization of images was obtained with Anatomist software (CEA NeuroSpin, Gif-sur-Yvette, France, http://brainvisa.info).

### Evaluation of the blood-brain barrier function

Evans Blue dye was diluted in saline solution and administered by retro-orbital injection in CDOX NEM290, CDOX NEM292 and Control non-xenografted mice at 100mg/kg (10ml/kg such as 100μl were injected per 10g of weight). Whole brains were harvested 30 minutes later and imaged under a fluorescent macroscope before they were sectioned in 1 mm thick slices in a brain matrix to precisely observe Evans blue dye extravasation (bright-field) and tumor infiltration (red fluorescence). As a positive control, peripheral organs were checked for their blue coloration. The kidney of the control mouse was harvested and imaged such as described above.

## SUPPLEMENTARY MATERIALS FIGURES AND TABLES




