# Supplementary material for: New in vivo avatars of diffuse intrinsic pontine gliomas (DIPG) from stereotactic biopsies performed at diagnosis
Source: Oncotarget. 2017 Feb 2;8(32):52543–59. doi: 10.18632/oncotarget.15002 (PMC5581049; doi:10.18632/oncotarget.15002)
Supplement: Supplementary file 1 [file oncotarget-08-52543-s001.pdf]

New *in vivo* avatars of diffuse intrinsic pontine gliomas (DIPG) from stereotactic biopsies performed at diagnosis

SUPPLEMENTARY MATERIALS

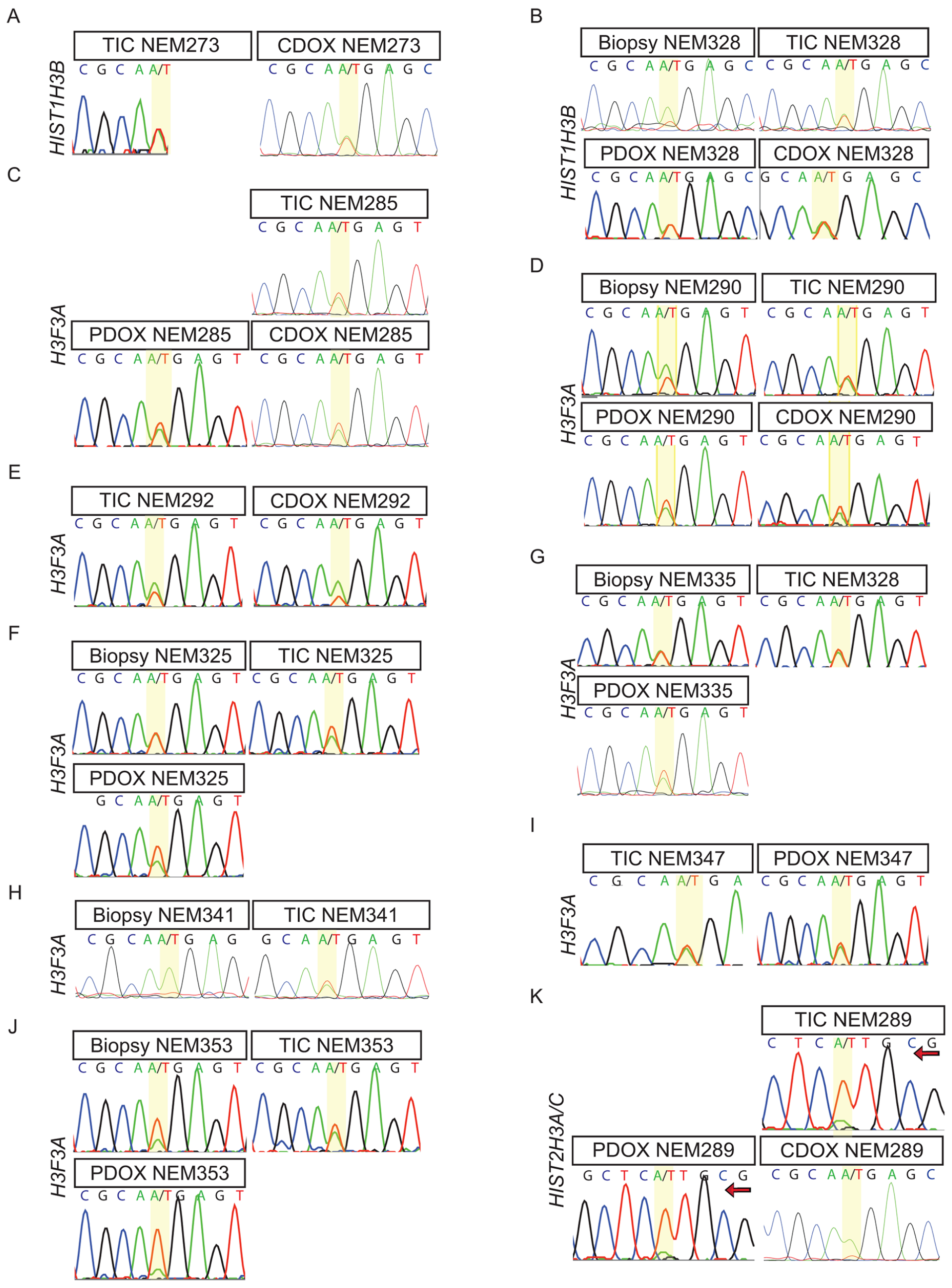

Supplementary Figure 1: Conservation of histone H3 mutation at position K27 in *in vitro* and *in vivo* DIPG models. Sanger sequencing of the *HIST1H3B* **A-B.**, *H3F3A* **C-J.** and *HIST2H3A/C* **K.** genes in the primary tumor and corresponding tumor initiating cells (TIC), PDOX and CDOX models. The A to T transversions are highlighted in yellow. The sequence alignments of Whole Exome Sequencing data of *HIST2H3A/C* (*Continued*)

L

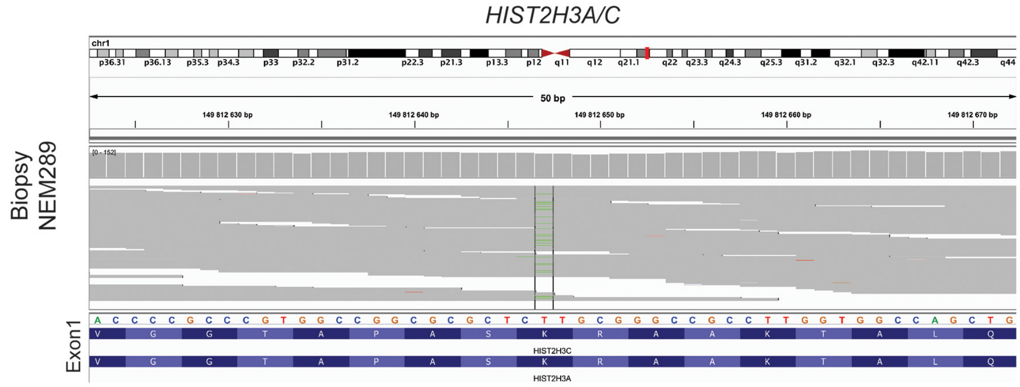

M

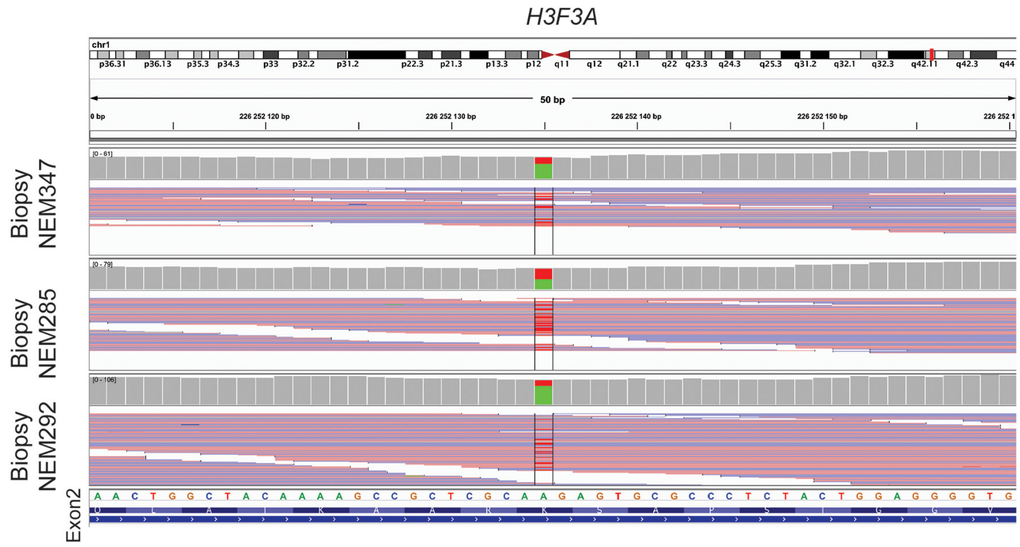

N

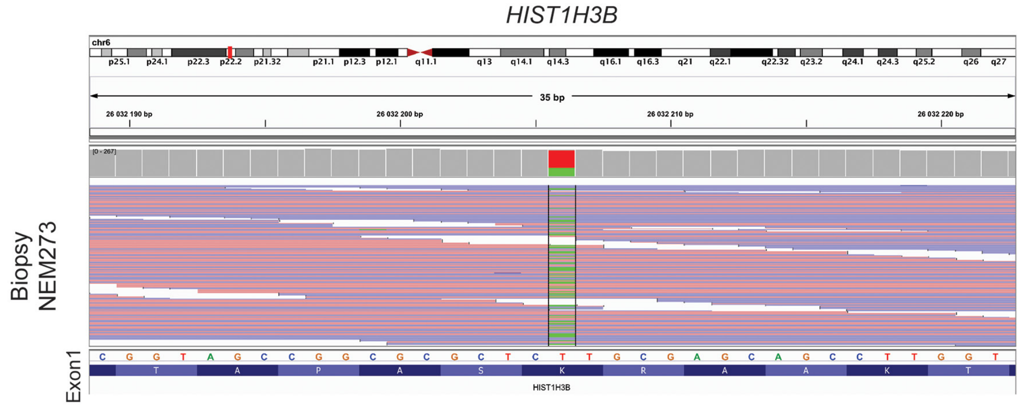

Supplementary Figure 1: (*Continued*) Conservation of histone H3 mutation at position K27 in *in vitro* and *in vivo* DIPG models. L., *H3F3A* **M.** and *HIST1H3B* **N.** genes were viewed using the Integrative Genomics Viewer (IGV) for 5 distinct biopsies. NEM289 biopsy is mutated in *HIST2H3A* at position K27 as shown by green bases. The variant allele frequency is estimated at 20% (reads appear in grey due to multiple alignment since the locus is duplicated in the genome). *H3F3A* K27M mutation was confirmed in NEM347, NEM285 and NEM292 biopsies as the mutation was found in reads at a frequency of 30%, 50% and 24%, respectively (wild-type and mutated bases are shown in green and red, respectively). *HIST1H3B* K27M was detected in 65 % of the reads (wild-type and mutated bases are shown in green and red, respectively).

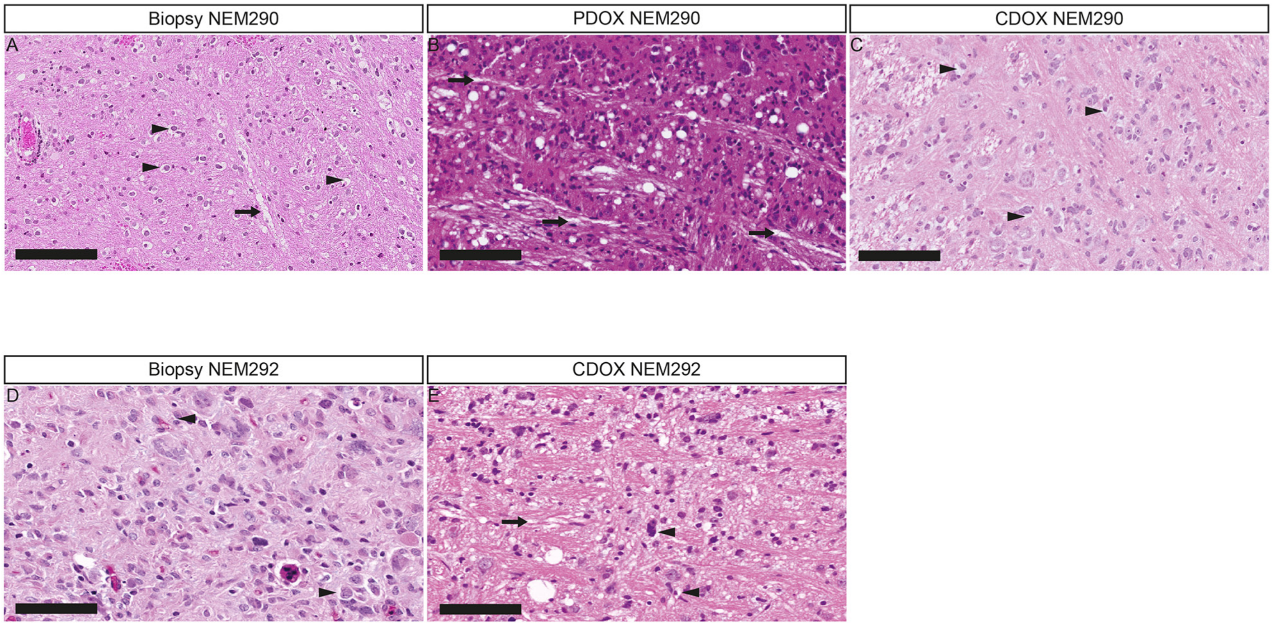

Supplementary Figure 2: Extracellular edema and edema along axonal stream in PDOX and CDOX models in regards to the primary tumor. Hematoxylin and Eosin coloration for NEM290 biopsy, PDOX NEM290 & CDOX NEM290 **A-C.**, and NEM292 biopsy & CDOX NEM292 **D and E.**, respectively. The three models harbor extracellular edema as observed in the primary tumors, even if more pronounced in the case of NEM292. Some edema following axonal streams is also observed in most cases. (Arrowheads indicate pericellular edema. Arrows indicate edema following axonal streams. Scale bars: 125 µm).

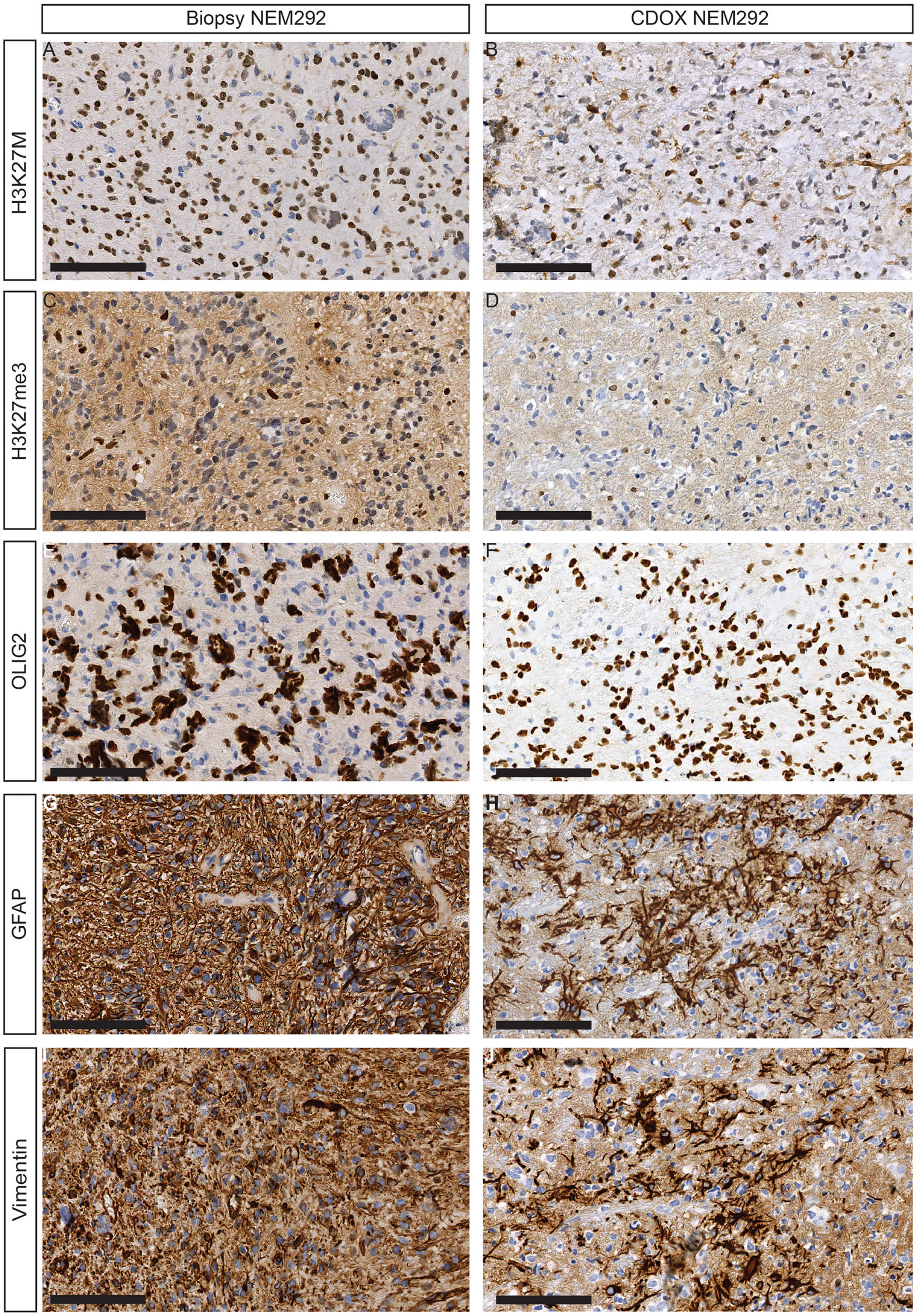

Supplementary Figure 3: CDOX NEM292 model displays DIPG hallmarks and consistent histopathological features with the tumor of origin. Representative results of immunohistochemistry for H3-K27M **A-B.**, H3K27me3 **C-D.**, OLIG2 **E-F.**, GFAP **G-H.** and Vimentin **I-J.** in the primary tumor biopsy NEM292 and sagittal sections of the corresponding CDOX model. The model recapitulates the histopathological profile of the human tumor of origin with the presence of infiltrative DIPG cells showing a loss of H3K27 trimethylation together with the expression of the mutated histone H3-K27M. OLIG2 detects most tumor cells in the pons, whereas Vimentin, GFAP and MIB-1 are only labelling a subset of the tumor compartment. As for NEM290 (cf. Figure3), the reactive astrogliosis is recapitulated with a strong detection of intermediate filament expression in mouse stromal cells. (Scale bars: 50 µm).

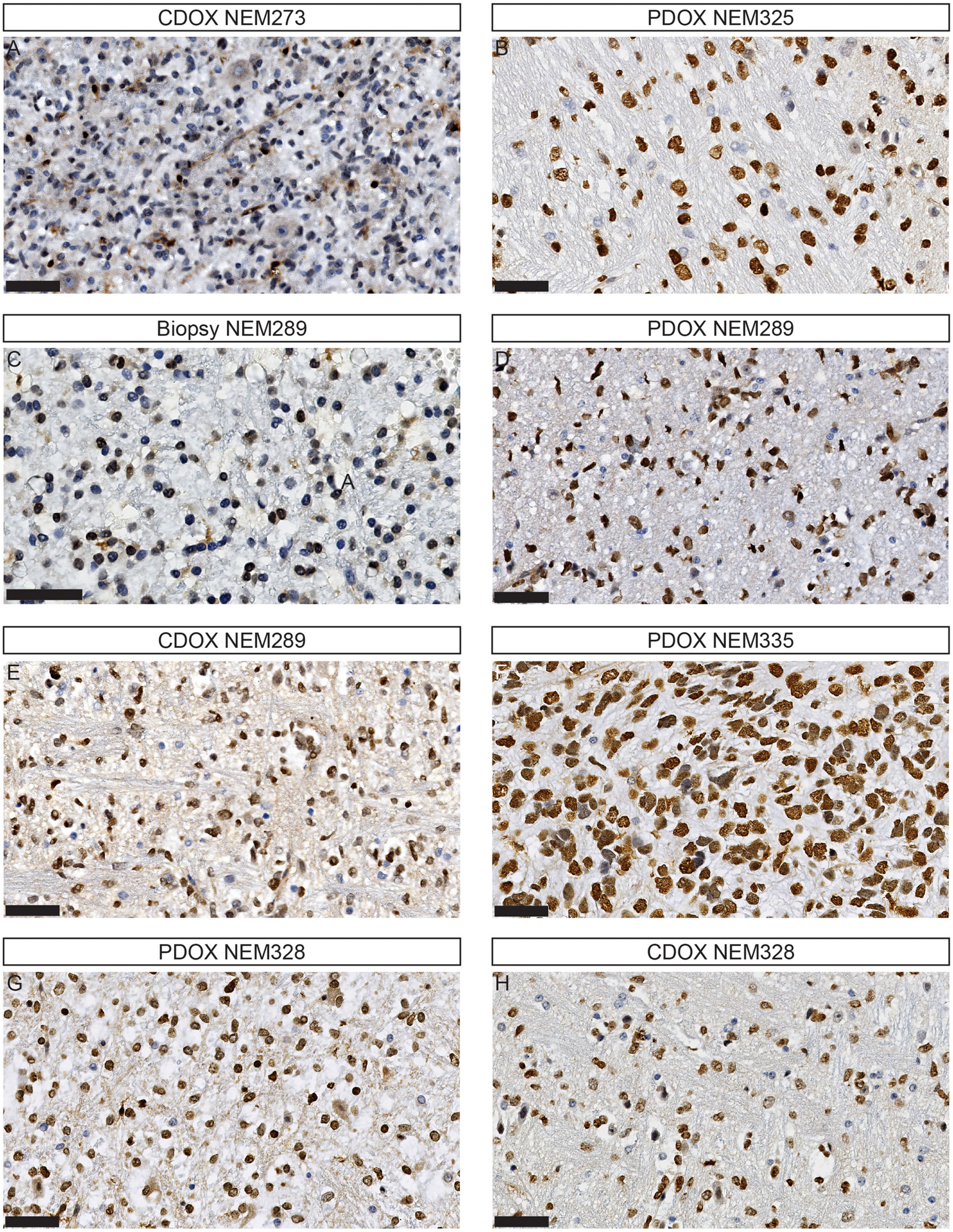

Supplementary Figure 4: CDOX and PDOX models retain H3-K27M mutation. Representative results of immunohistochemistry for H3-K27M (brown nuclei) for 9 CDOX/PDOX models and 3 corresponding biopsies (when available). Scale bars indicate 50 µm. (*Continued*)

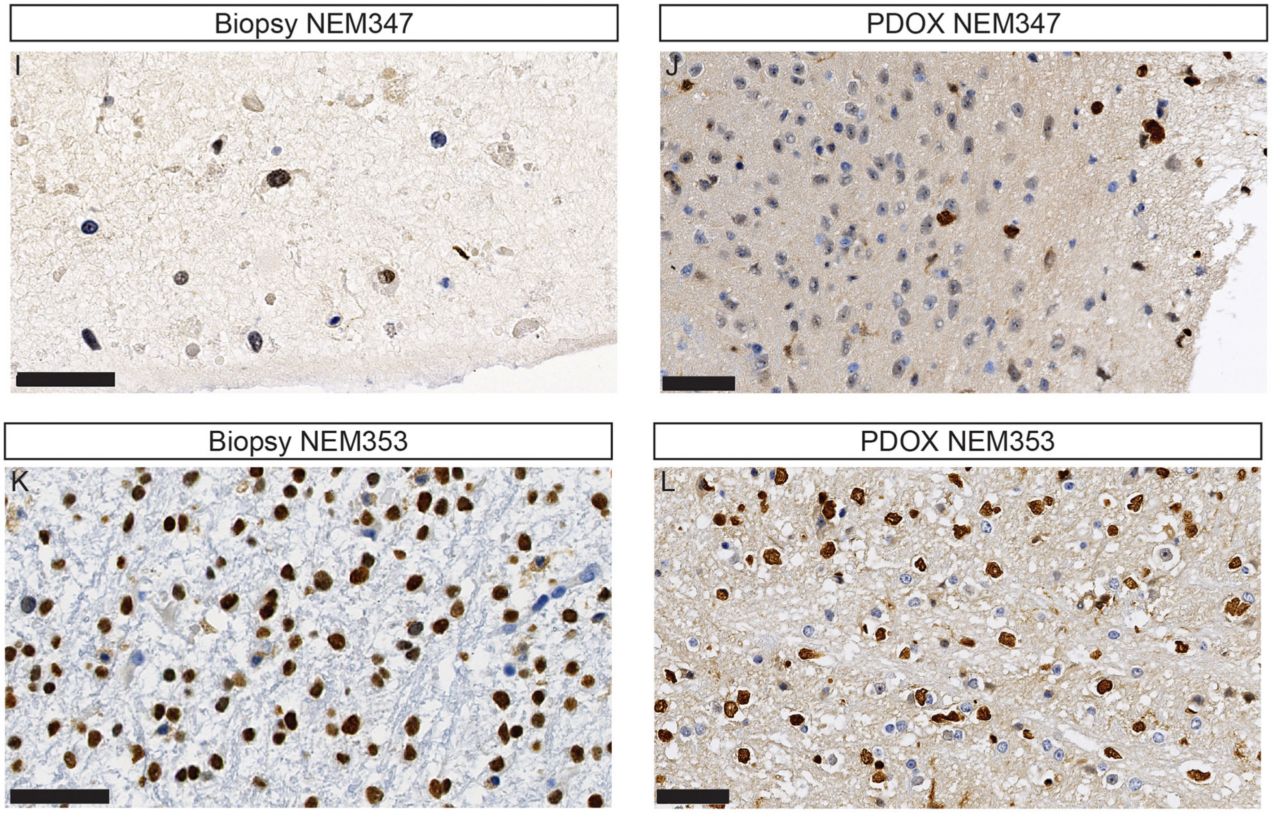

Supplementary Figure 4: (*Continued*) CDOX and PDOX models retain H3-K27M mutation. Representative results of immunohistochemistry for H3-K27M (brown nuclei) for 9 CDOX/PDOX models and 3 corresponding biopsies (when available). Scale bars indicate 50 µm.

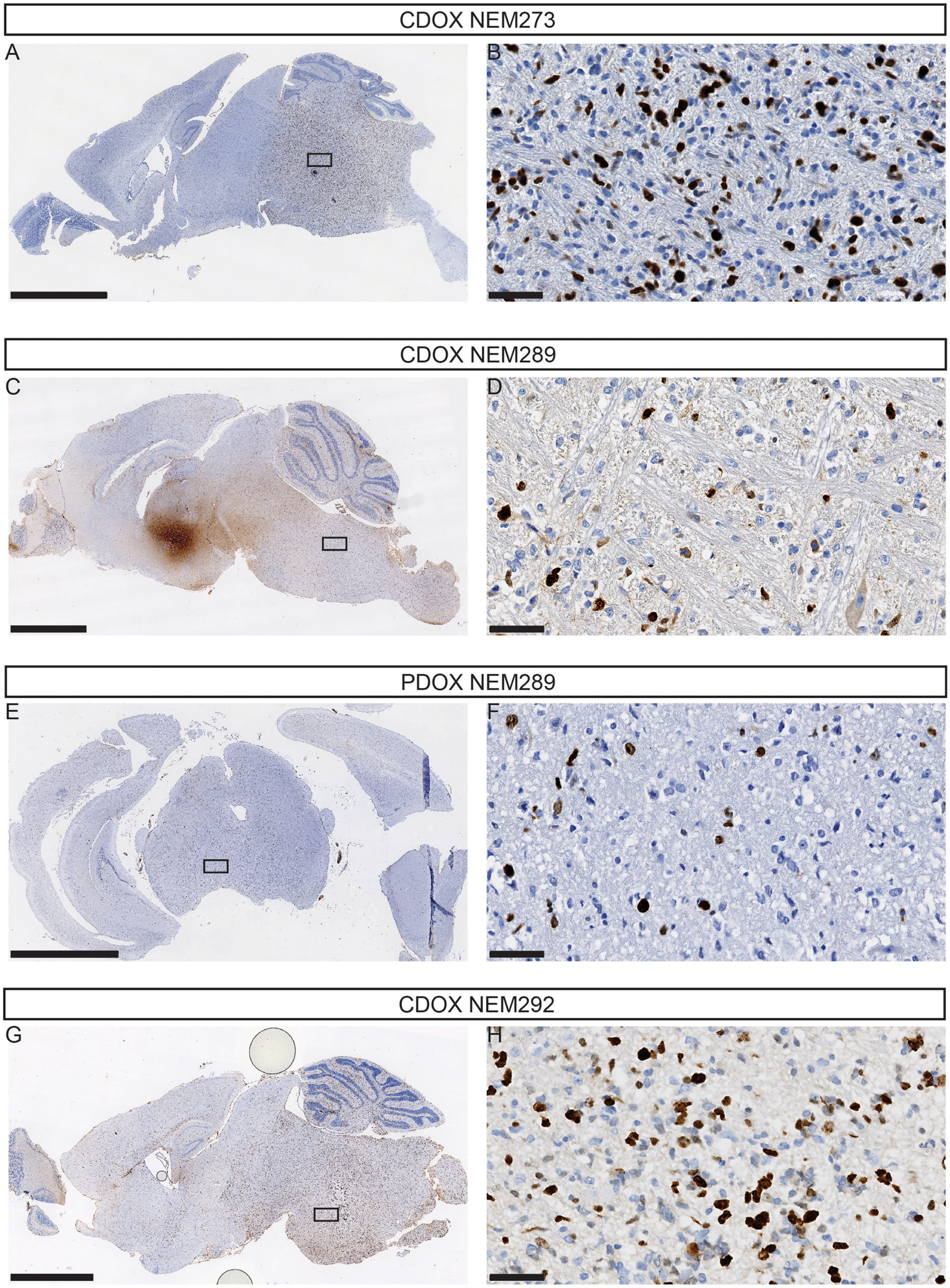

Supplementary Figure 5: MIB-1 staining identifies proliferating cells of human origin in DIPG xenografts. Left: Full sagittal sections of brains stained for MIB-1 (brown nuclei) are represented for 8 distinct xenografts **A, C, E, G** (*Continued*)

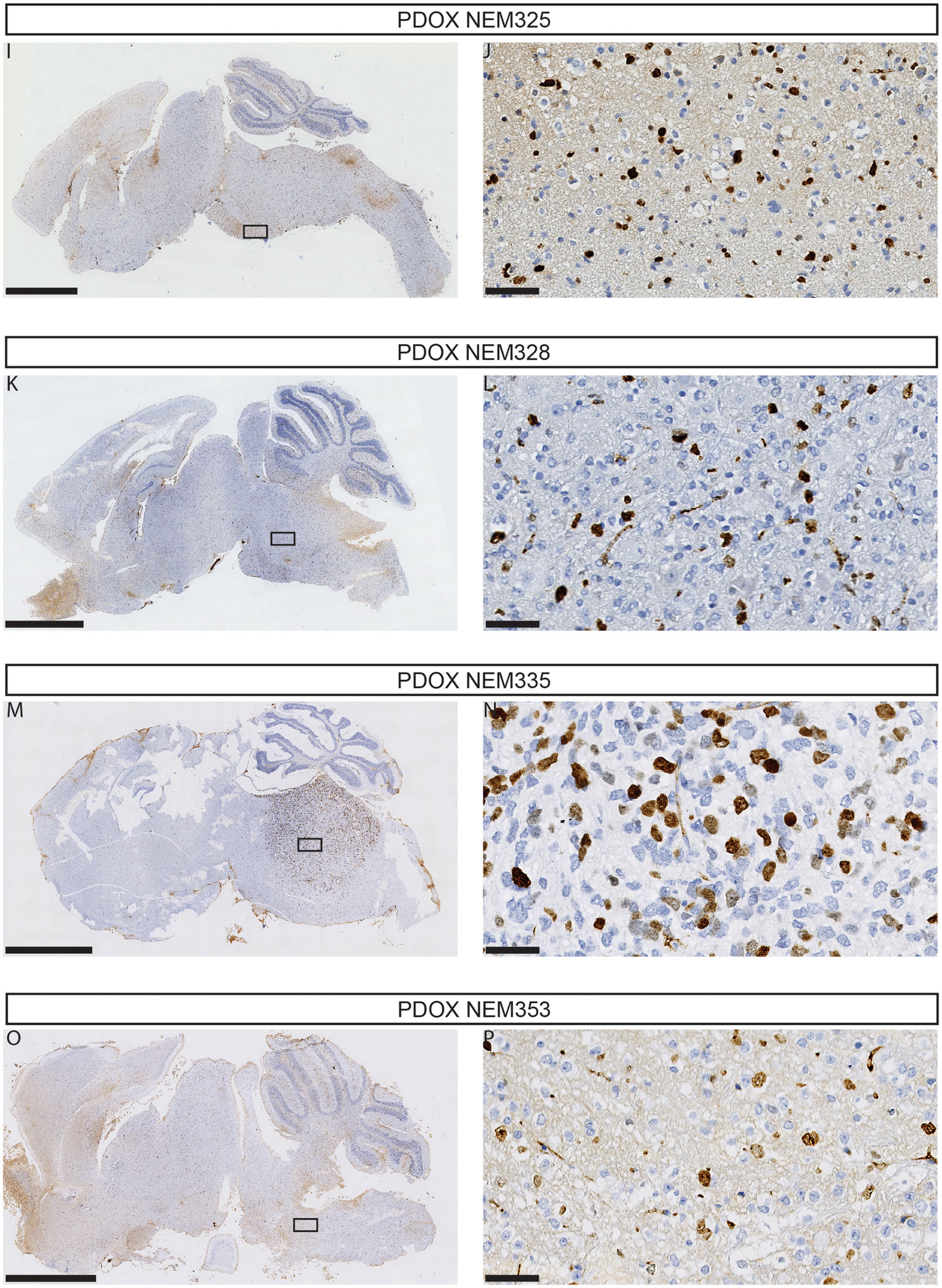

Supplementary Figure 5: (*Continued*) MIB-1 staining identifies proliferating cells of human origin in DIPG xenografts. **I, K, M, and O.** Right: 40X magnification of the square regions highlighted in the corresponding left panels **B, D, F, H, J, L, N, and P.** Scale bars indicate 50 µm.

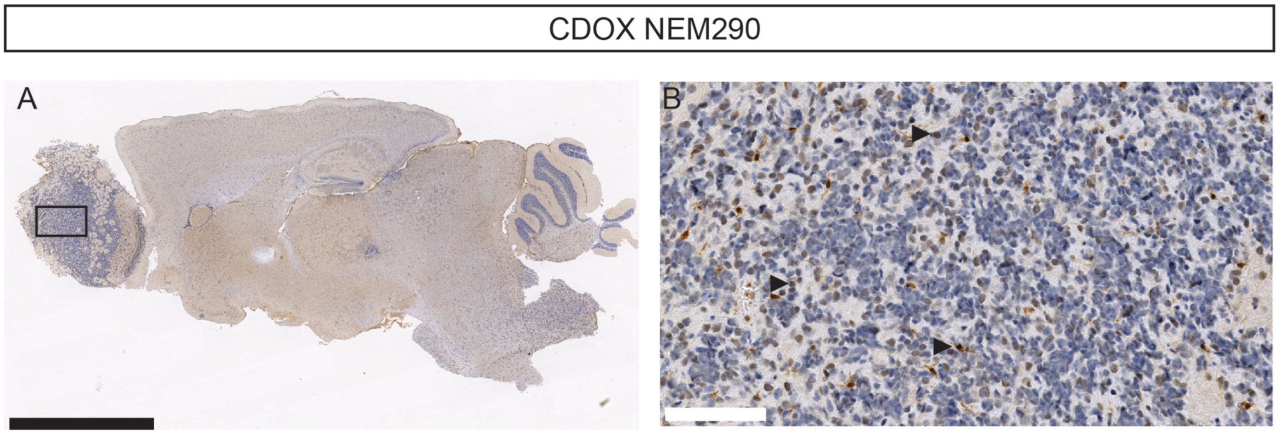

Supplementary Figure 6: Extensive DIPG cells invasion toward the olfactory bulbs in CDOX NEM290. **A.** Full sagittal view of H3-K27M staining in CDOX NEM290 model (brown nuclei) and **B.** Olfactory bulb region corresponding to a 20X magnification of the highlighted rectangle in **A**. Arrowheads indicate tumor cells. Scale bars indicate 2.5 mm and 100 µm, respectively.

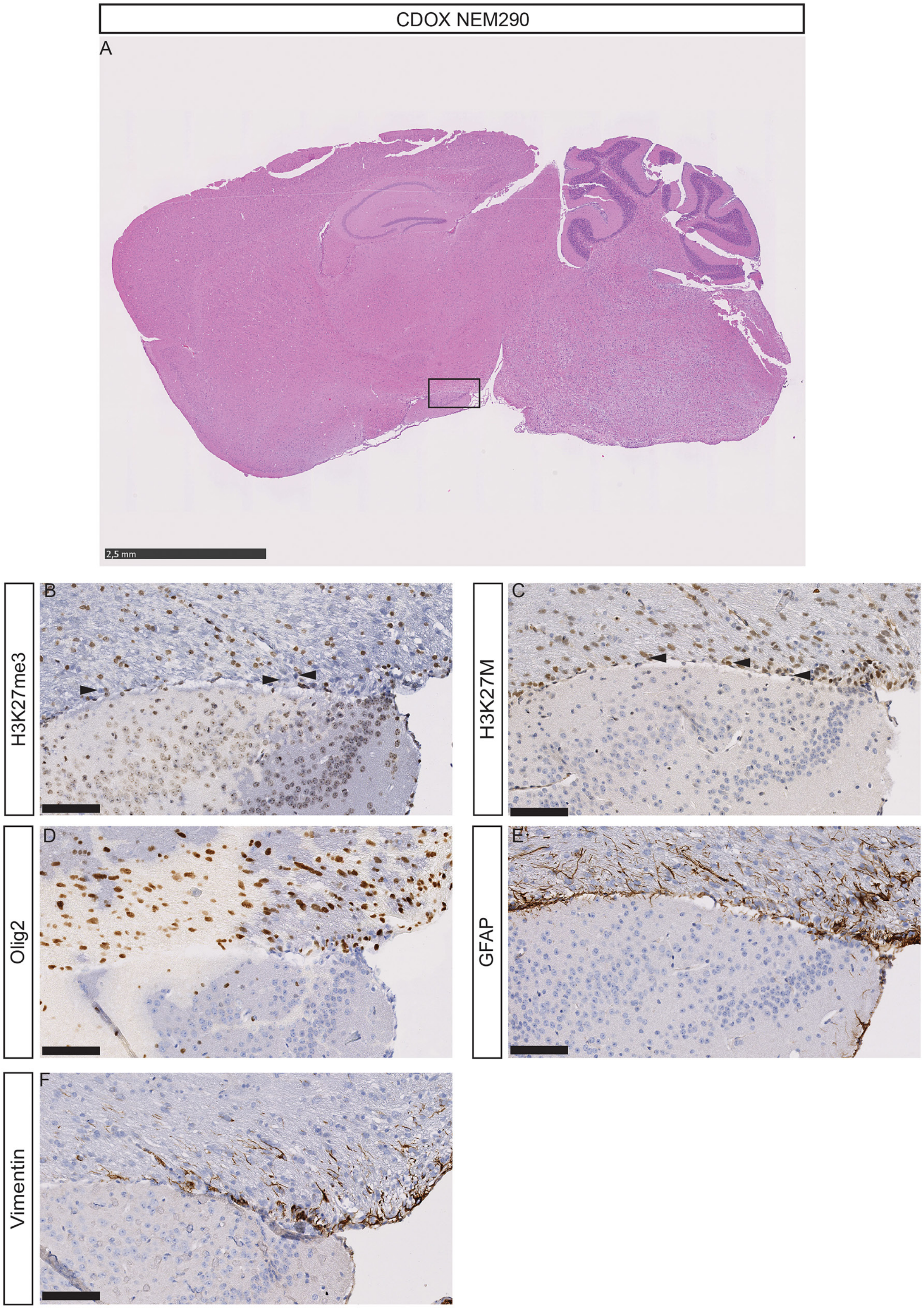

Supplementary Figure 7: Extensive infiltration in the sub-ventricular zone and cerebral peduncles in CDOX NEM290. **A.** H&E coloration of a sagittal section of CDOX NEM290. The rectangle represents the zoomed area shown in **B-F** delimiting the sub-ventricular region and the cerebral peduncle. Staining for **B.** H3K27me3 with arrowheads indicating cells with a H3K27me3 loss, **C.** H3-K27M with arrowheads indicate mutated tumor cells, **D.** OLIG2, **E.** GFAP and **F.** Vimentin. (Scale bars 100 µm).

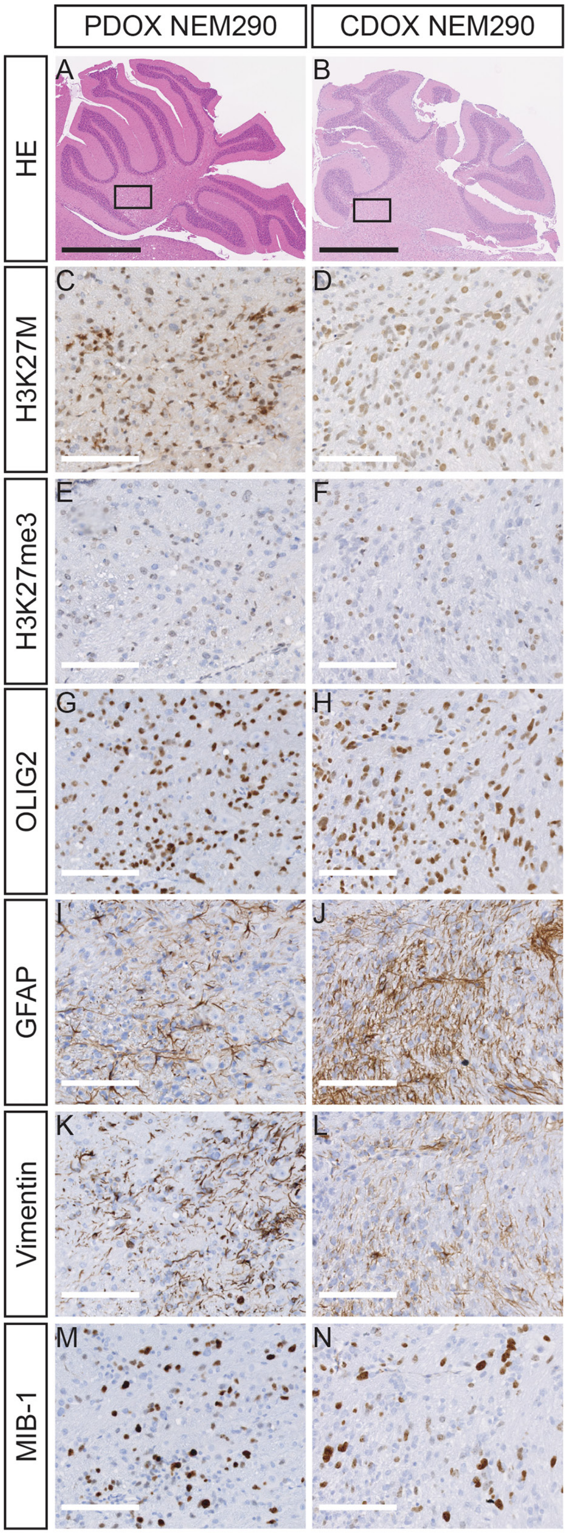

Supplementary Figure 8: Extensive infiltration in the cerebellum in PDOX and CDOX NEM290 models. **A-B.** H&E coloration of the cerebellar region of PDOX and CDOX NEM290 models. Scale bars indicate 1 mm. Black rectangles represent the zoomed area for the immunohistochemistry staining presented below: **C-D.** H3-K27M, **E-F.** H3K27me3, **G-H.** OLIG2, **I-J.** GFAP, **K-L.** Vimentin, and **M-N.** MIB-1 of PDOX and CDOX NEM290 models, respectively. (Scale bars 125 µm).

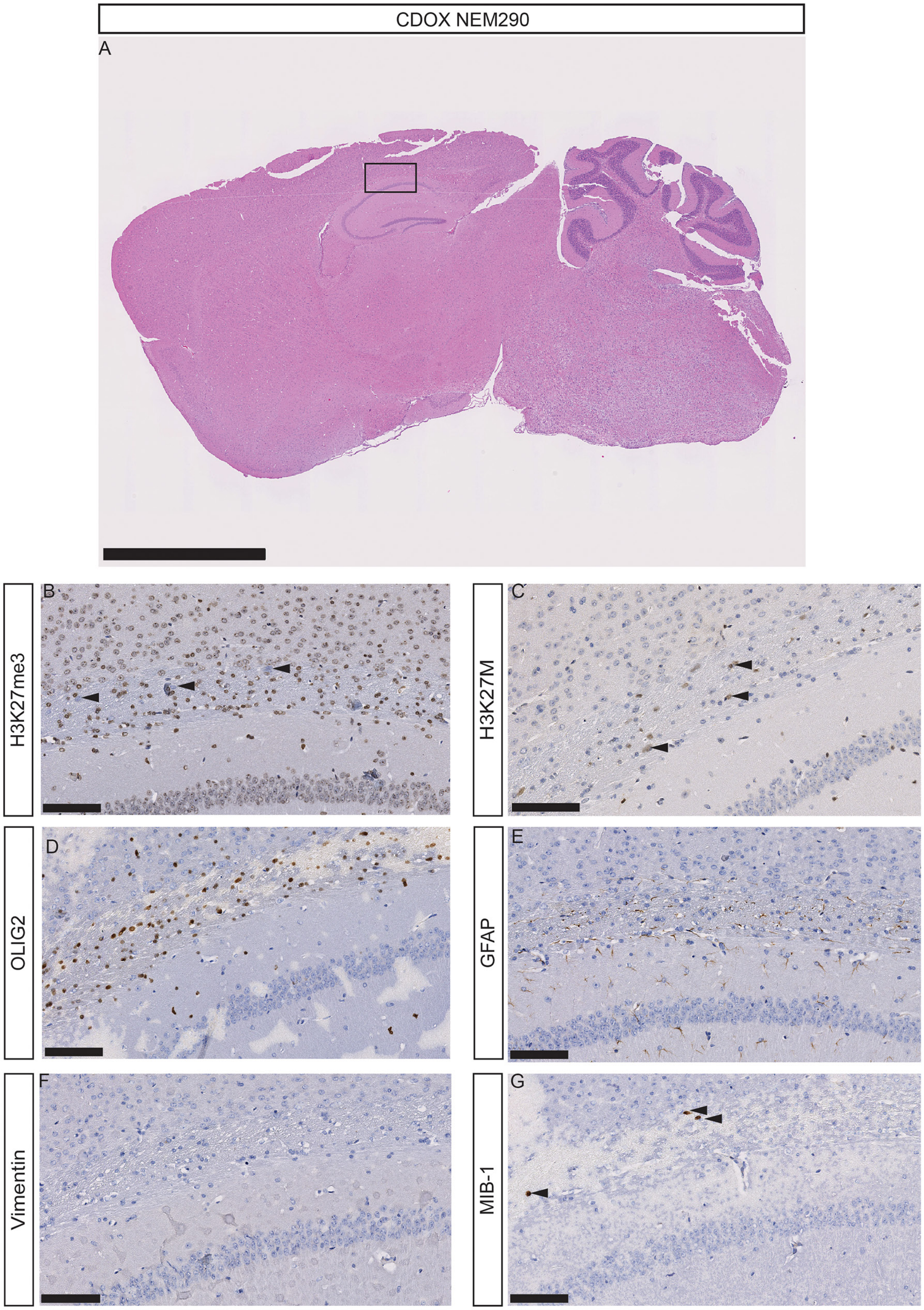

Supplementary Figure 9: Extensive infiltration in the neocortex in CDOX NEM290. A. H&E coloration of a full sagittal section of CDOX NEM290. Scale bar indicates 2.5 mm. The rectangle represents the cortical zoomed area presented in B-G panels. Staining for **B.** H3K27me3 – arrowheads indicate cells with a H3K27me3 loss; **C.** H3-K27M, arrowheads indicate tumor cells; **D.** OLIG2; **E.** GFAP; **F.** Vimentin; and **G.** MIB-1, arrowheads indicate positive tumor cells. (Scale bars: 100 µm).

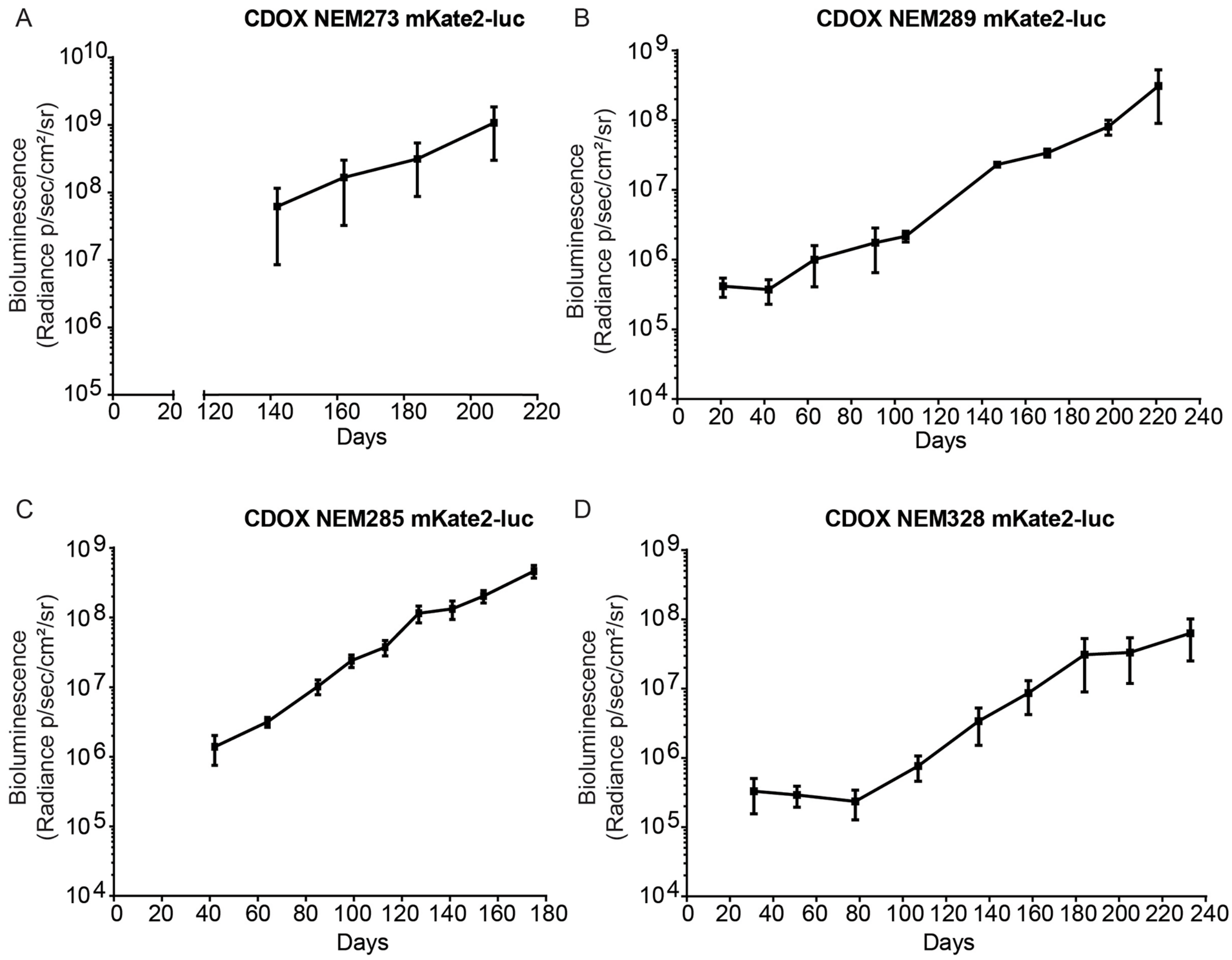

Supplementary Figure 10: Tumor growth monitoring by bioluminescence Longitudinal follow-up of tumor growth by quantification of the luciferase activity in the pontine region of 4 distinct CDOX models. As shown in Figure 5A-5B for NEM290 and NEM292, all CDOX models presented a robust exponential tumor growth. Results are represented as mean ±SEM.

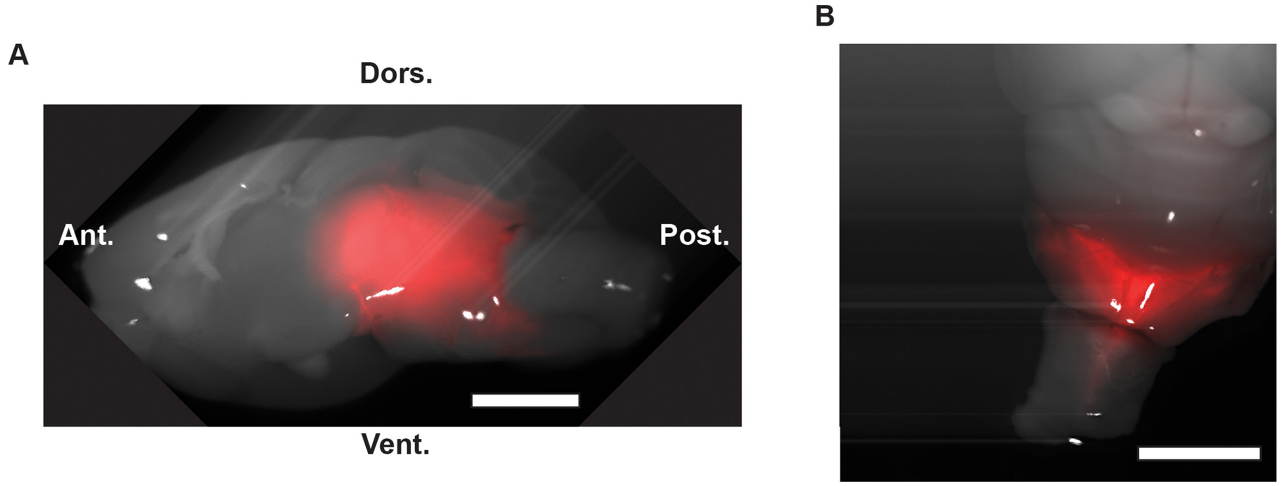

Supplementary Figure 11: Whole brain imaging by macroscopy allows the *ex-vivo* detection of tumor. A. Lateral and **B.** Upright view of CDOX NEM290 whole brain showing tumor location with red fluorescence. (Scale bars: 1 cm).

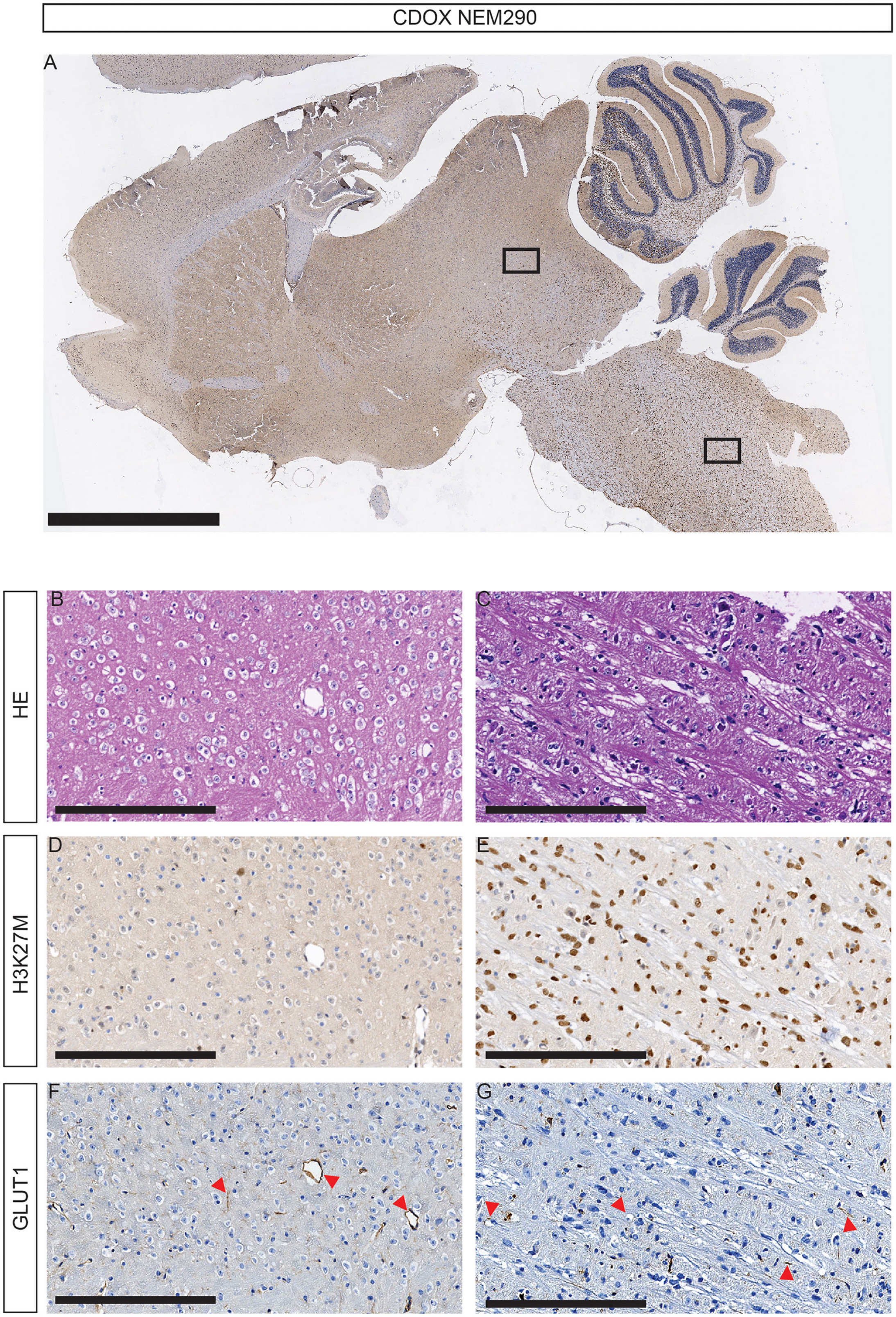

Supplementary Figure 12: GLUT1 expression in CDOX NEM290 and CDOX NEM292 mouse models. A. Image of a full sagittal section of CDOX NEM290 stained for H3-K27M. Rectangles represent the regions detailed in **B-G**. 30X magnification microphotographs of **B.** H&E, **D.** H3-K27M and **F.** GLUT1 staining in a brain area with few tumor cells positive for H3-K27M, and corresponding microphotograph of **C.** H&E, **E.** H3-K27M and **G.** GLUT1 staining in the infiltrated pons (*Continued*)

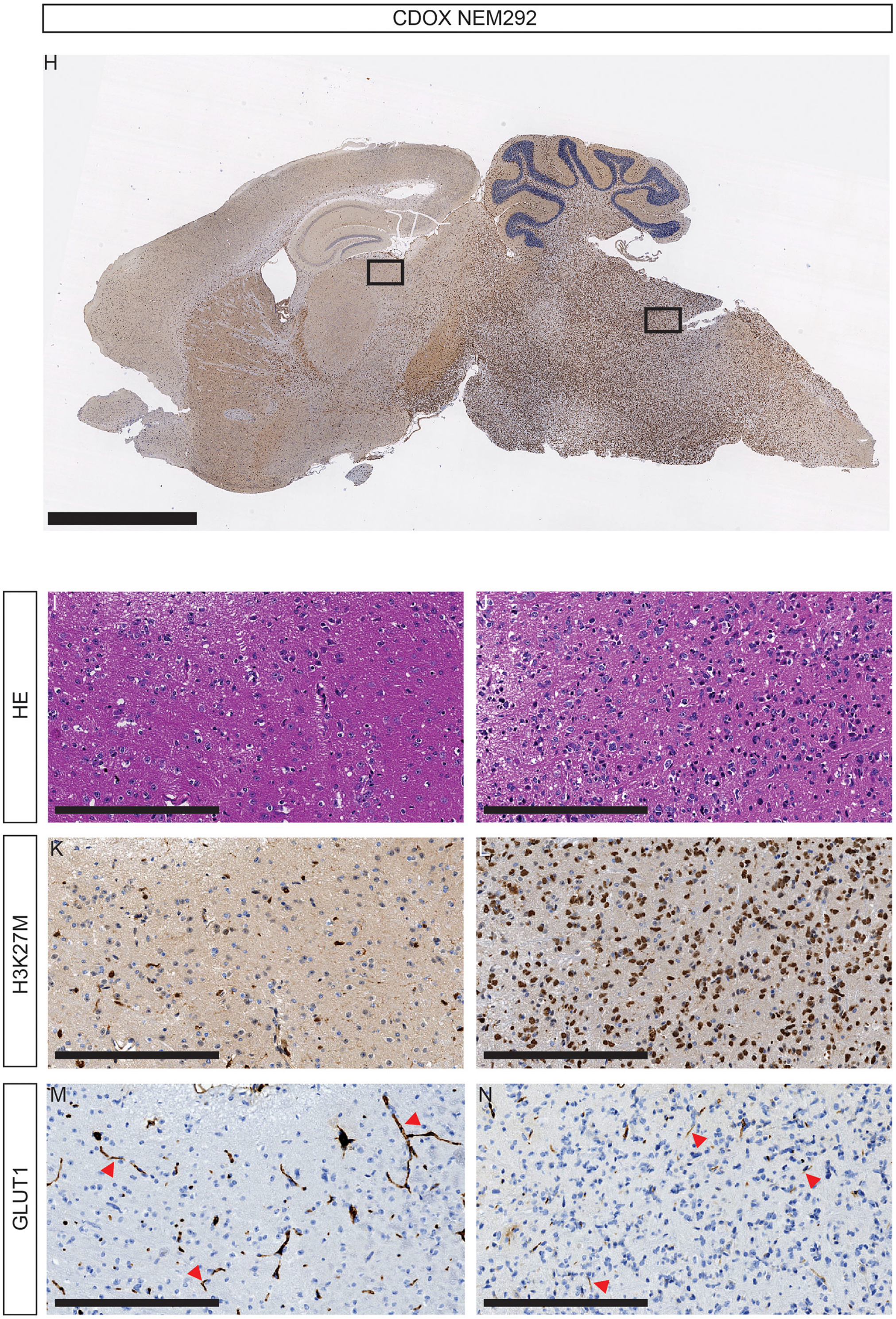

Supplementary Figure 12: (*Continued*) GLUT1 expression in CDOX NEM290 and CDOX NEM292 mouse models. **H-N.** identical staining and magnification in CDOX NEM292. There was no overt microvascular proliferation observed in tumors **C, J.** despite an important proportion of tumor cells presenting a brown K27M detection **E, L.** GLUT1 expression was strongly detected in endothelial cells from vessel and capillaries in the normal brain **F, M.**, but vessels were sparser and showing slightly fainter staining in the bulk of the tumor. Arrowheads indicate examples of GLUT1 expressing endothelial cells. (Scale: 2.5 mm in A, H ; 250 µm in other panels).

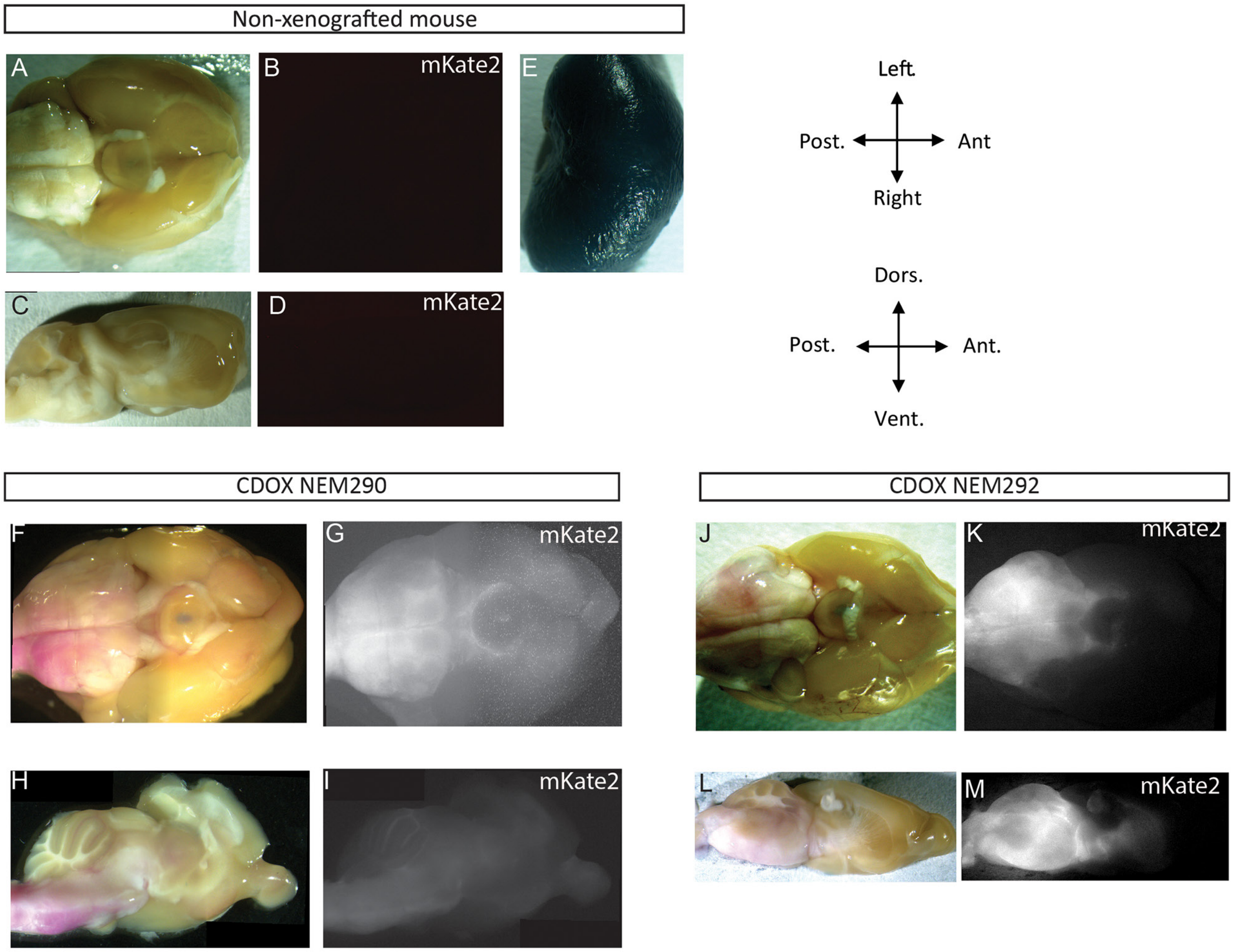

Supplementary Figure 13: NEM290 and NEM292 CDOX models display a functional blood-brain barrier. A, F, and J. Macroscopic reconstitution of whole brains of a non-injected control mouse and NEM290 and NEM292 CDOX models after Evans blue dye intravenous injection, and **B, G, and K.** corresponding mKate2 epifluorescence as recorded by fluorescence-macroscopy. **E.** Kidney was used as positive control of Evans blue dye extravasation as exemplified for a control non-xenografted mouse. **C, H, and L.** Bright field microphotograph of whole sagittal sections of the brains depicted in previous panels and **D, I, and M.** corresponding mKate2 red-fluorescence detection. As in control mouse, there was no detection of blue staining in bright-field images, that would underlie an extravasation of the Evans blue dye in the grafted mice despite an important infiltration of the pontine region by tumor cells (mKate2 red fluorescence). This pointed therefore to an overall functional blood-brain barrier in the DIPG xenograft.

Supplementary Table 1: Detailed engraftment rate and survival time of serially transplanted mice (PDOX).

For each PDOX model, the number of mice presenting tumors over the total number of injected mice was detailed according to passage number and also to the location of the injection (pons vs. thalamus). The average survival time of mice that developed a tumor at each passage are indicated in days. When cells were frozen prior to the transplant, the term "postfreezing" was added in the header, and survival tested vs. non-frozen samples (*P-value<0.05, T-test).

Survival data are expressed as mean ±SD. SD was not displayed when all mice reached end-point the same day. N.D., Not determined.

Supplementary File 1

Supplementary Table 2: Short-Tandem Repeat (STR) DNA profiling of primary tumors and xenograft models.

Patient ID	MARKER	D1S1656	D6S1043	Penta E	D2S1338	D8S1179
**NEM273**	**Biopsy**	13, 15	12, 19	11, 13	23, 25	13, 14
	**CDOX**	13, 15	12, 19	11, 13	23, 25	13, 14
**NEM285**	**Biopsy**	11, 15	11, 11	10, 13	18, 23	13, 14
	**PDOX**	11, 15	11, 11	10, 13	18, 23	13, 14
	**CDOX**	11, 15	11, 11	10, 13	18, 23	13, 14
**NEM289**	**Biopsy**	11, 15.3	12, 12	5, 14	23, 25	10, 11
	**PDOX**	11, 15.3	12, 12	5, 14	23, 25	10, 11
	**CDOX**	11, 15.3	12, 12	5, 14	23, 25	10, 11
**NEM290**	**Biopsy**	12, 14	13, 19	10, 11	17, 23	10, 14
	**PDOX**	12, 14	13, 19	10, 11	17, 23	10, 14
	**CDOX**	12, 14	13, 19	10, 11	17, 23	10, 14
**NEM292**	**Biopsy**	15.3, 17	10, 17	11, 12	22, 24	10, 15
	**CDOX**	15.3, 17	10, 14	11, 12	22, 24	10, 15
**NEM325**	**Biopsy**	15, 17.3	12, 20	10, 13	19, 24	13, 13
	**PDOX**	15, 17.3	12, 20	10, 13	19, 24	13, 13
**NEM328**	**Biopsy**	16, 16.3	13, 13	13, 13	17, 18	11, 11
	**PDOX**	16, 16.3	13, 13	13, 13	17, 18	11, 11
	**CDOX**	16, 16.3	13, 13	13, 13	17, 18	11, 11
**NEM335**	**Biopsy**	14, 16.3	11, 12	5, 7	18, 24	12, 13
	**PDOX**	14, 16.3	11, 12	5, 7	18, 24	12, 13
**NEM347**	**Biopsy**	12, 12	11, 12	12, 12	17, 23	10, 12
	**PDOX**	12, 12	11, 12	12, 12	17, 23	10, 12
**NEM353**	**Biopsy**	12, 15	14, 20	7, 11	17, 24	12, 14
	**PDOX**	12, 15	14, 20	7, 11	17, 24	12, 14

The STR profile on 5 selected loci is presented for all tested samples and confronted to patient blood DNA used as reference.
