# Supplementary material for: New in vivo avatars of diffuse intrinsic pontine gliomas (DIPG) from stereotactic biopsies performed at diagnosis
Source: Oncotarget. 2017 Feb 2;8(32):52543–59. doi: 10.18632/oncotarget.15002 (PMC5581049; doi:10.18632/oncotarget.15002)
Supplement: Supplementary file 2 [file oncotarget-08-52543-s002.docx]

| **Model ID** | **Location** | **P0** | | **P1** | | **P1 Post-freezing** | | **P2** | | **P3** | | **P4** | | **P4 Post-freezing** | | **P5** | |
| --- | --- | --- | --- | --- | --- | --- | --- | --- | --- | --- | --- | --- | --- | --- | --- | --- | --- |
|  |  | **Engraft. rate** | **Survival (days)** | **Engraft. rate** | **Survival (days)** | **Engraft. rate** | **Survival (days)** | **Engraft. rate** | **Survival (days)** | **Engraft. rate** | **Survival (days)** | **Engraft. rate** | **Survival (days)** | **Engraft. rate** | **Survival (days)** | **Engraft. rate** | **Survival (days)** |
| **PDOX NEM285** | Thalamus | 2/2 | 169 | - | - | 4/5 | 216 ±10 * | 10/10 | 163 ±17 | *-* | *-* | *-* | *-* | *-* | *-* | *-* | *-* |
|  | Pons | *-* | *-* | 3/3 | 123 ±6 | *-* | *-* | *-* | *-* | *-* | *-* | *-* | *-* | *-* | *-* | *-* | *-* |
| **PDOX NEM289** | Thalamus | 2/2 | 142 | - | - | 4/5 | 144 ±10 | 12/12 | 140 ±22 | 5/5 | 118 | *-* | *-* | *-* | *-* | *-* | *-* |
|  | Pons | *-* | *-* | 3/3 | 105 ±18 | *-* | *-* | *-* | *-* | 5/5 | 100 ±4 | *-* | *-* | *-* | *-* | *-* | *-* |
| **PDOX NEM290** | Thalamus | 2/2 | 171 ±5 | 9/9 | 89 ±8 | 5/5 | 145 ±8 * | 9/9 | 81 ±7 | 9/9 | 90 ±2 | *-* | *-* | 5/6 | 110 ±16 * | *-* | *-* |
|  | Pons | *-* | *-* | *-* | *-* | *-* | *-* | *-* | *-* | 3/3 | 81 ±3 | *-* | *-* | 4/4 | 74 | 3/5 | 87 ±20 |
| **PDOX NEM325** | Thalamus | 2/2 | 294 | 2/5 | 288 | N.D/2 | N.D | N.D/3 | N.D | *-* | *-* | *-* | *-* | *-* | *-* | *-* | *-* |
|  | Pons | 1/1 | 145 | 5/5 | 299 ±12 | 3/3 | 308 * | N.D/2 | N.D | *-* | *-* | *-* | *-* | *-* | *-* | *-* | *-* |
| **PDOX NEM328** | Thalamus | 2/2 | 274 | 5/5 | 201 ±23 | *-* | *-* | 4/5 | 182 ±21 | N.D/3 | N.D | *-* | *-* | *-* | *-* | *-* | *-* |
|  | Pons | 1/1 | 223 | 5/5 | 157 ±8 | *-* | *-* | 3/6 | 215 ±16 | N.D/2 | N.D | *-* | *-* | *-* | *-* | *-* | *-* |
| **PDOX NEM335** | Thalamus | 2/2 | 163 | 2/3 | 150 | *-* | *-* | 4/5 | 165 ±3 | 3/3 | 75 ±6 | *-* | *-* | *-* | *-* | *-* | *-* |
|  | Pons | 1/1 | 139 | 1/2 | 133 | *-* | *-* | 1/3 | 127 | *-* | *-* | N.D/5 | N.D | *-* | *-* | *-* | *-* |
| **PDOX NEM347** | Thalamus | 3/3 | 155 | 2/2 | 123 | *-* | *-* | N.D/3 | N.D | N.D/2 | N.D | *-* | *-* | *-* | *-* | *-* | *-* |
|  | Pons | 2/2 | 145 ±31 | 2/2 | 121 ±4 | *-* | *-* | N.D/2 | N.D | N.D/2 | N.D | *-* | *-* | *-* | *-* | *-* | *-* |
| **PDOX NEM353** | Thalamus | 2/2 | 224 | N.D/3 | N.D | *-* | *-* | *-* | *-* | *-* | *-* | *-* | *-* | *-* | *-* | *-* | *-* |
|  | Pons | 1/1 | 81 | N.D/2 | N.D | *-* | *-* | *-* | *-* | *-* | *-* | *-* | *-* | *-* | *-* | *-* | *-* |
